# The novel HRD motif kinase SPE-60 is required for sperm development and motility in *Caenorhabditis elegans*

**DOI:** 10.1038/s41598-025-18696-2

**Published:** 2025-09-15

**Authors:** Dieter-Christian Gottschling, Frank Döring

**Affiliations:** Department of Molecular Prevention, University of Kiel, 24118 Kiel, Germany

**Keywords:** Caenorhabditis elegans, Developmental biology, Genetic interaction, Mutation, Cell biology

## Abstract

Sexual reproduction requires sperm development, activation and motility. Many of these post-meiotic processes remain incompletely understood. We identified the gene *spe-60*, which encodes a tau-tubulin kinase with a conserved catalytic HRD motif. Localisation via immunostaining reveals that SPE-60 is present in sperm and dynamically colocalises with membranous organelles (MOs), that store factors essential for activation and fertility. *spe-60* null mutants are sterile due to immotile sperm that undergo normal MO-plasma membrane fusion but fail to extend normal pseudopods essential for motility and fertilisation. Furthermore, mutant sperm exhibit arrested spermatocyte development. The functional loss observed in *spe-60* null sperm is phenocopied by sperm carrying a CRISPR/Cas-mediated point mutation within the HRD motif. Genetic epistasis places SPE-60 downstream of the serine/threonine kinase SPE-6 in the canonical sperm activation pathway. Thus, SPE-60 is required for sperm development and motility, but not MO fusion. These findings suggest that the HRD-containing kinase SPE-60 fulfils a dual function as a key evolutionarily conserved factor.

**Supplementary Information:**

The online version contains supplementary material available at 10.1038/s41598-025-18696-2.

## Introduction

Fertilisation is a fundamental event for the survival of species, and spermatogenesis plays a central role in its success. A key component of sexual reproduction is the production of motile spermatozoa capable of oocyte fertilisation and zygote formation. This complex process involves a cascade of coordinated events, including spermatocyte differentiation, spermatid activation, directional movement, oocyte contact, and gamete fusion^1–5^. Defects in spermatogenic proteins, particularly kinases, phosphatases, and proteases, frequently cause sterility across many species, including mammals, insects, and nematodes^6–9^.

In mammals, protein kinases regulate sperm motility, capacitation, and immune interactions^10–12^. Tau-tubulin kinases, such as TTBK1 or its orthologs, are involved in the regulation of post-meiotic sperm development and motile cilia formation^13^ and play a pivotal role in the pathogenesis of neurodegenerative diseases^14^, thus underscoring the broader physiological significance of these enzymes. In sea urchins, sperm release into seawater triggers an increase in intracellular pH, stimulating motility and acrosome reactions via cAMP-dependent pathways^15,16^. In insects, particularly *Drosophila melanogaster*, serine/threonine kinases (dTSSKs), orthologous to mammalian TSSKs, are indispensable for male fertility, influencing both sperm storage and competitive success^17–19^. Studies in nematodes, such as *Ascaris suum* and *Caenorhabditis elegans*, demonstrate that sperm-enriched transcripts and proteins are disproportionately composed of kinases, phosphatases, and ion-binding proteins, underscoring the central role of phosphorylation in sperm development^20–23^. Specifically in *C. elegans*, microarray and proteomic analyses reveal that kinases and phosphatases are significantly overrepresented in sperm compared with oocytes, reinforcing the idea that dynamic phosphorylation events are fundamental to sperm differentiation and function^24,25^. Despite these insights, the full regulatory landscape of sperm biology, particularly the mechanisms governing sperm activation, remains incompletely understood.

The nematode *C. elegans* is a widely used model organism for developmental and molecular studies^26–28^. It exhibits two sexes: hermaphrodites and males. In both, the overall process of sperm cell development (spermatogenesis) takes place during the L4 larval stage and involves two sequential phases. The first of these encompasses the meiotic differentiation of spermatocytes into spermatids, while the second comprises post-meiotic differentiation (spermiogenesis), which includes the activation and functional maturation of spermatids into motile spermatozoa capable of fertilisation^29,30^. While spermatocyte differentiation into spermatids is conserved across sexes, spermiogenesis is context dependent. In hermaphrodites, spermatid activation is induced instantly by physiological cues within the reproductive tract. In contrast, in males, spermatids remain quiescent until transferred to a mating hermaphrodite. This implies that the stimuli responsible for the initial activation signal may differ between hermaphrodites and males. The molecular control of spermiogenesis is tightly regulated by the SPE-8 pathway in both hermaphrodites and males, comprising several transmembrane proteins (SPE-12, SPE-19, SPE-27, SPE-29) and the kinase SPE-8, which act to inhibit premature activation by repressing the downstream casein kinase SPE-6^31–33^. In the male reproductive system, sperm activation is regulated by the seminal protease TRY-5 and its inhibitor, SWM-1. While both the SPE-8 and TRY-5 pathways are functional in males, only the SPE-8 pathway has been demonstrated to be functional in hermaphrodites^24,34^. Upon appropriate stimulation, two key cellular events define sperm activation: (1) fusion of membranous organelles (MOs) with the plasma membrane (PM), and (2) extension of a pseudopod, enabling amoeboid motility^6,35,36^. These events are functionally similar to mammalian capacitation and acrosome reaction, which prepare sperm for fertilisation^37,38^. The process of MO fusion is similar to the acrosome reaction with regard to cytological features and biological significance.

Notably, the pseudopod, which contains MO-derived fertilisation factors on its surface, is the site where spermatozoa interact with oocytes^36,39^. In *C. elegans*, spermatozoa use a unique crawling motility powered by a major sperm protein (MSP) cytoskeleton, distinct from actin-based systems in other species^40,41^. The regulated assembly and disassembly of MSP filaments is critical for movement and has been linked to kinase-driven posttranslational modifications in the proteins involved^23,42,43^. Moreover, the pseudopod is the location where interaction between the spermatozoon and the oocyte takes place.

Despite the progresses made, the identity and mechanisms of many kinases regulating these sperm-specific events remain unclear. Kinases typically contain a conserved catalytic core, some of which including a HRD motif within the catalytic loop, which is critical for positioning substrates, catalyzing phosphate transfer, and allostery^44–46^. The HRD motif—comprising histidine (H), arginine (R), and aspartate (D)—is evolutionarily conserved and functionally essential in diverse eukaryotic protein kinases that undergo allosteric regulation. In *Drosophila*, the HRD motif of SRC64 kinase is required for cytoskeletal functions and fertility, while in humans, HRD-containing kinases such as Janus kinases (JAKs) and the Abelson kinase (Abl) play central roles in cellular signaling, with implications in oncogenesis and immune regulation^47,48^.

Each residue of the HRD motif contributes distinct functions. HRD-*Asp* acts as a catalytic base, orienting substrates for phosphoryl transfer. Its mutation typically abolishes kinase activity. HRD-*Arg*, though less conserved across kinases, mediates allosteric communication between regulatory and catalytic domains, and its presence is a hallmark of allosterically regulated kinases^49,50^. In contrast, the role of HRD-*His* is less defined and requires further investigation^51^.

In this study, we identify *spe-60*, a novel member of the *spe* (spermatogenesis-defective) gene family in *C. elegans*, encoding a previously uncharacterised HRD motif-containing tau-tubulin protein kinase also found in neurons^52^. We demonstrate that *spe-60* is essential for both sperm development (spermatogenesis) and function (spermiogenesis). Moreover, we show that a specific amino acid substitution in the catalytic HRD motif is sufficient to abolish kinase activity in SPE-60, leading to profound defects in meiotic differentiation and sperm motility, resulting in sterility in both males and hermaphrodites. These findings establish SPE-60 as a critical regulator of sperm development and function and highlight the functional significance of the HRD motif in kinase signaling.

## Results

### *spe-60* encodes a sperm-associated kinase required for hermaphrodite self-fertility

**Fig. 1:**
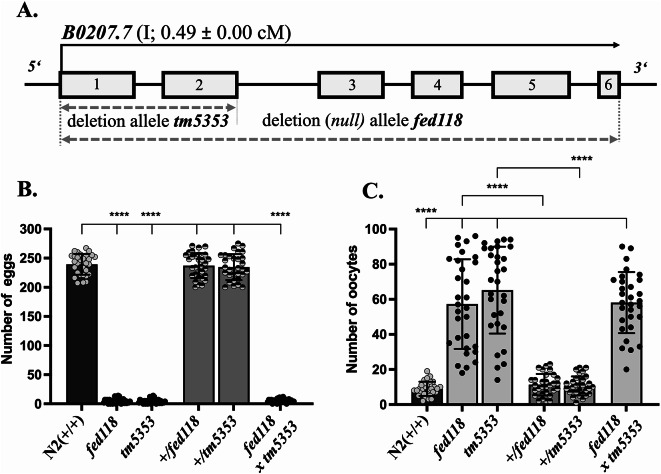
The loss of *B0207.7* disrupts hermaphrodite self-fertility. **(A)** Schematic representation of the *B0207.7* gene structure. Exons are depicted as numbered boxes (1–6). Dashed arrows indicate the regions deleted in the *tm5353* and the CRISPR/Cas9 generated *fed118* deletion alleles. **(B**,** C)** Quantification of egg **(B)** and oocyte **(C)** numbers produced by N2 wild-type (+/+), heterozygous (+/–), homozygous (–/–) *fed118* and *tm5353* mutants, as well as trans-heterozygous *tm5353*/*fed118* hermaphrodites. Heterozygous animals were generated by crossing mutant hermaphrodites with mutant or wild-type males. Data represent the means (± SD) of *N* = 3 independent experiments involving *n* ≥ 10 animals per trial. *p < 0.0332; **p < 0.0021; ***p < 0.0002; ****p < 0.0001 (ordinary one-way ANOVA).

In order to identify novel regulators of reproduction, we performed a series of forward and in silico reverse genetic screens targeting candidate genes implicated in fertility. These led to the identification of B0207.7 through its STRING^53^ interaction with another novel spermatogenic protein, which is currently being examined in order to determine its role in fertility. The gene *B0207.7* is located on chromosome I (0.49 ± 0.00 cM), encoding a putative serine/threonine tau-tubulin kinase of 373 amino acids (Fig. 1A). High-resolution spatial transcriptomic profiling, including RNA tomography, revealed a pronounced enrichment of *B0207.7* transcripts in sperm, with expression dynamics mirroring those of canonical *spe* (SPErmatogenesis defective) genes^39,54,55^. Accordingly, in the present study, *B0207.7* is referred to as *spe-60*.

Functional analysis using the deficiency allele *tm5353* - harbouring a deletion including exons 1 and 2 of the *spe-60* genomic sequence - demonstrated that *spe-60(tm5353)* hermaphrodites were self-sterile, producing almost exclusively unfertilised oocytes compared to wild-type animals that predominantly produce fertilised eggs (Fig. S1). Furthermore, the B0207.7 deficiency was not found to induce embryonic-lethal phenotypes (Fig. S3). Mutant animals laid an average of 65 ± 25 unfertilised oocytes, a marked increase relative to wild-type controls (9 ± 4) and exhibited a dramatically reduced self-brood size (0–14 progeny vs. 239 ± 18 in wild type). (Fig. 1B, C)

To validate these observations, we generated the CRISPR/Cas9-derived *null* allele *fed118*, which deletes the complete *spe-60* genomic locus. Homozygous *spe-60(fed118)* animals phenocopy the *spe-60(tm5353)* mutants, displaying drastically reduced egg counts (4 ± 4) and elevated oocyte numbers (61 ± 26). In contrast, heterozygous animals (+/*tm5353* and +/*fed118*) exhibited normal reproductive output, indicating that both are recessive *null* alleles. Furthermore, *tm5353*/*fed118* trans-heterozygotes were indistinguishable from the corresponding homozygous mutants (Fig. 1B, C), reinforcing the conclusion that *spe-60* is indispensable for hermaphrodite self-fertility. In this study, the deletion allele *spe-60(fed118)* was referred to as *spe-60(null)*.

Together, these findings demonstrate that *spe-60* encodes a sperm-associated protein kinase essential for the self-fertility of hermaphrodites. Its loss disrupts fertilisation, resulting in sterility characterised by a dramatically reduced number of eggs an excessive number of unfertilised oocytes.

### A single amino acid substitution in the catalytic HRD motif of SPE-60 disrupts fertility in hermaphrodites

**Fig. 2:**
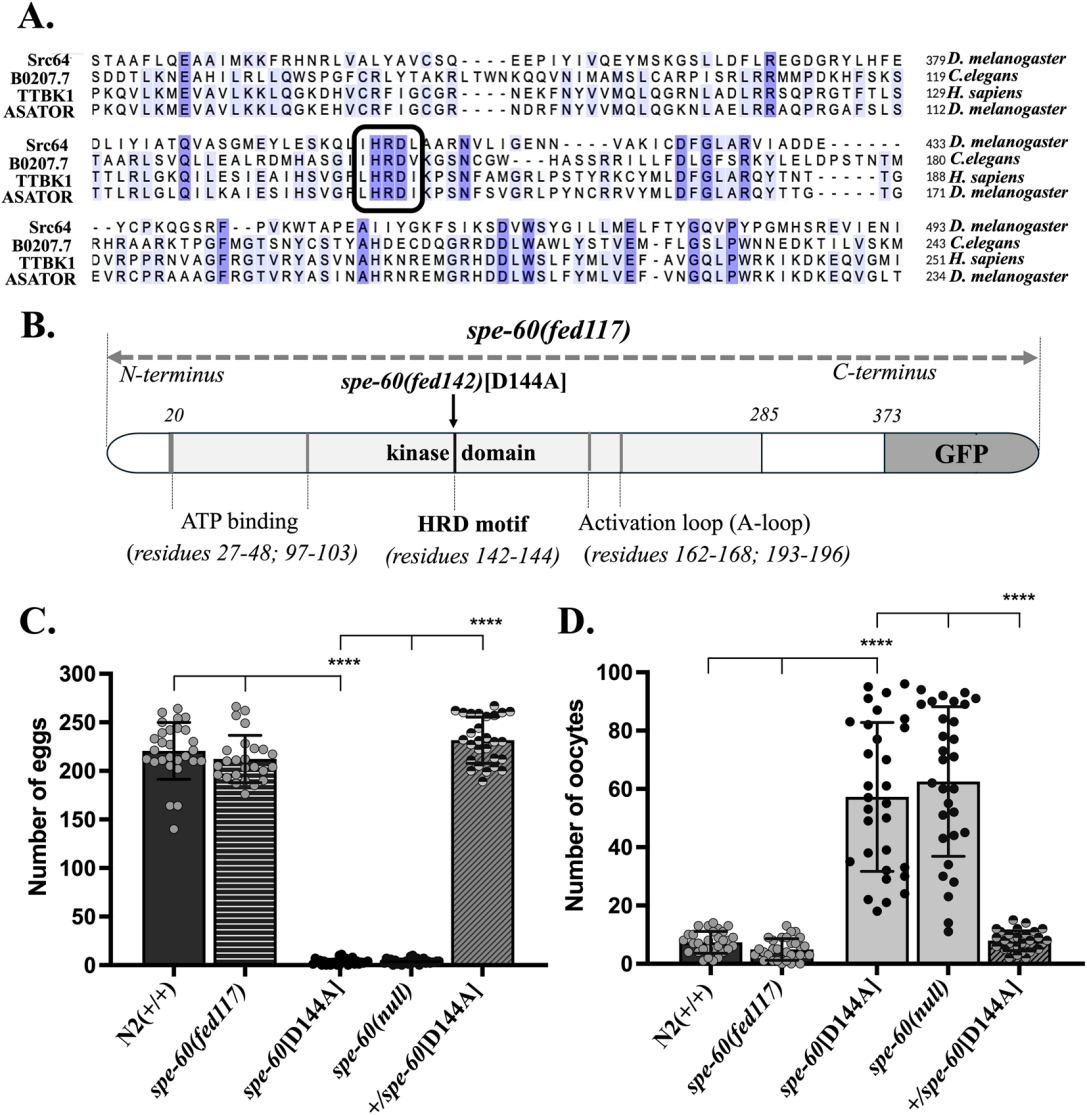
The catalytic HRD motif of the SPE-60 kinase is required for hermaphrodite self-fertility. **(A)** Kinase domain sequence alignment between kinases with conserved HRD motif from human (TTBK1), *D. melanogaster* (Src64, Asator), and *C. elegans* (B0207.7 (SPE-60)) showing sequence similarities marked in blue. The conserved catalytic loop HRD motifs are highlighted with the black square. **(B)** Schematic illustration of the SPE-60 protein which shows the predicted kinase domain (bright grey), including a N-terminal ATP binding sites (mid grey), the ATP- and substrate binding HRD motif (black), and the activation loop (dark grey). The conserved *His-Arg-Asp* residues of the HRD motif are known to sustain kinase activity, as they are involved in the allosteric coupling and the transfer of a phosphate group of ATP to a bound substrate. The arrow indicates the amino acid exchange D (*Asp*) to A (*Ala*) at the position 144 in the *fed142* allele. The numerical values represent the positions of the first (residue 20) and the last (residue 285) amino acid residue of the predicted kinase domain. The number of eggs **(C)** and unfertilised oocytes **(D)** laid by mutants carrying the D144A exchange in comparison to N2 wild-type (+/+) worms, *spe-60(null)* mutants, and heterozygous *+/spe-60*[D144A]. The *+/spe-60*[D144A] animals represent the F1 progeny resulting from the crossing of homozygous *spe-60*[D144A] worms with the N2 wild types (+/+). The values represent the means (± SD) of *N* = 3 independent experiments involving *n* ≥ 10 animals per trial. *p < 0.0332; **p < 0.0021; ***p < 0.0002; ****p < 0.0001 (ordinary one-way ANOVA).

Building on the identification of *spe-60* as a putative serine/threonine kinase essential for hermaphrodite self-fertility, we sought to dissect the structural domains underpinning its enzymatic function. In silico functional proteomics, employing the *InterPro* resource^56^ and the *NCBI* Conserved Domain Database^57^revealed that *spe-60* shares sequence similarity with tau-tubulin kinases of the casein kinases superfamily and harbours a canonical N-terminal serine/threonine kinase domain spanning the amino acid residues 20–285. This conserved domain encompasses amino-acid ATP-binding motifs (residues 27–48, 97–103), a HRD catalytic motif (residues 142–144) which is a hallmark of allosterically regulated kinases^47,50^, and an activation loop (residues 162–168, 193–196) (Fig. 2A, B).

To question the functional relevance of the HRD motif in SPE-60, we generated a CRISPR/Cas9-derived recessive loss-of-function allele at the native *spe-60* genomic locus, *fed142*, introducing an aspartate-to-alanine Substitution at position 144 (D144A) within the motif, and a C-terminal green fluorescent protein (GFP) fusion (Fig. 2B). We labeled this allele as *spe-60*[D144A]. Strikingly, *spe-60*[D144A] homozygous hermaphrodites exhibited a significant reduction in fertility, phenocopying the sterility observed in animals carrying the alleles *tm5353* and *spe-60(null)*. On average, [D144A] mutants produced only 5 ± 3 fertilised eggs and 67 ± 11 unfertilised oocytes, compared to 239 ± 18 eggs and 9 ± 4 oocytes in wild-type controls (Fig. 2C, D). Heterozygous F1 progeny resulting from [D144A] hermaphrodites crossed with wild-type males were fully fertile, indicating that the D144A substitution is recessive (Fig. 2C, D).

These findings establish that the aspartate residue within the HRD catalytic triad of *spe-60* is indispensable for its in vivo function. The loss of kinase activity mirrors the sterility phenotype of the *null* allele, underscoring the critical role of this catalytic motif in mediating fertility in hermaphrodites.

### SPE-60 is essential for male fertility and is required for sperm function

A substantial body of evidence indicates that sterility in *Caenorhabditis elegans* frequently arises from defects in sperm development and/or function. Given that *spe-60(null)* mutant hermaphrodites exhibit a markedly reduced brood size, elevated numbers of unfertilised oocytes, and that *spe-60* transcripts are highly enriched in sperm, we hypothesised that the observed sterility stems from altered sperm function. As hermaphrodite-derived sperm are required to support self-fertility in wild-type animals, we reasoned that male *spe-60(null)* mutants - producing only sperm - would display the same impaired fertility as the *spe-60(null)* hermaphrodites.

**Fig. 3:**
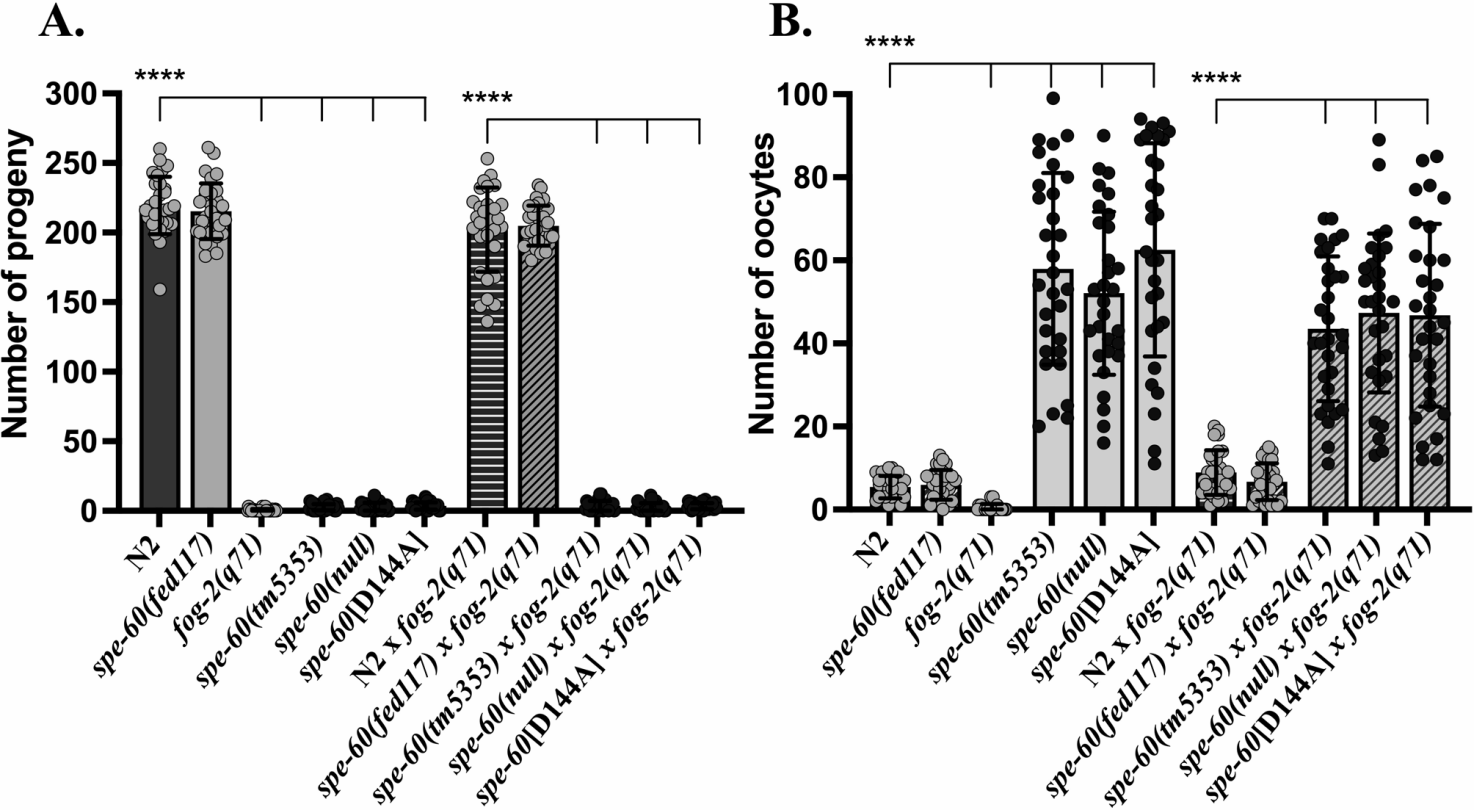
*spe-60* deficiency disrupts male fertility. **(A**,** B)** Quantification of eggs **(A)** and unfertilized oocytes **(B)** laid by self-fertilised N2 wild-type hermaphrodites and *fog-2(q71)* spermless hermaphrodites, in comparison to *fog-2(q71)* hermaphrodites mated with N2 wild-type males or males carrying the *spe-60* alleles *tm5353*,* null*, or *D144A*). Mating efficiency and male fertility were assessed by the ability of males to restore oocyte fertilisation in spermless hermaphrodites. The data represent the means (± SD) of *N* = 3 independent experiments involving *n* ≥ 10 animals per trial. *p < 0.0332; **p < 0.0021; ***p < 0.0002; ****p < 0.0001 (ordinary one-way ANOVA).

To test this, we performed mating assays using *spe-60(null)* males and *fog-2(q71)* hermaphrodites, which lack endogenous sperm and serve as a stringent test for male fertility. Strikingly, *spe-60* mutant males failed to fertilise *fog-2(q71)* animals, producing only 3 ± 3 eggs and 45 ± 19 unfertilised oocytes. In contrast, wild-type males successfully rescued fertility in *fog-2(q71)* hermaphrodites, yielding 202 ± 30 eggs and only 9 ± 5 unfertilised oocytes under identical conditions (Fig. 3A, B).

These data establish that *spe-60* is essential for male fertility. They also support the view that the sterility observed in hermaphrodites and males is due to identical phenotypes arising from defective sperm that are unable to fertilise eggs regularly.

### The SPE-60 kinase is dynamically present throughout all stages of sperm development

Given the essential role of SPE-60 in fertility, we next sought to determine its spatial and temporal distribution during sperm development. To visualise the endogenous localisation of the protein, we generated a CRISPR/Cas9-derived transgenic wild-type strain *spe-60(fed117)* expressing a C-terminally GFP-tagged SPE-60 fusion protein from the native *spe-60* locus under the control of its endogenous promoter.

**Fig. 4:**
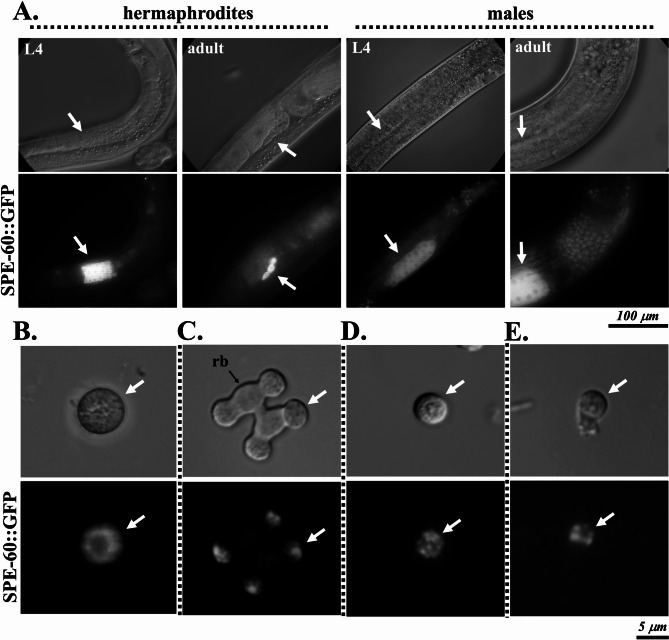
Cellular localisation of SPE-60 during distinct stages of sperm development. Differential interference contrast (DIC) and in vivo fluorescence images showing the expression and localisation pattern of SPE-60 in *spe-60(fed117)* worms expressing SPE-60::GFP fusion from the native *spe-60* genomic locus. **(A)** Whole-animal images of L4 and adult *spe-60(fed117)* worms displaying SPE-60::GFP (white arrows). **(B–E)** High-resolution images of individual sperm cells isolated from males at key stages of sperm development: primary spermatocytes **(B)**, secondary spermatocytes with residual body (rb) formation **(C)**, spermatids **(D)**, and mature spermatozoa **(E)**. White arrows indicate specific localisation sites of the SPE-60::GFP fusion within the respective cell types.

Sperm from *spe-60(fed117)* worms (expressing *spe60*p::*spe-60*::*gfp*) were phenotypically indistinguishable from N2 wild-type sperm (Figs. 2, 3 and 4). Confocal fluorescence in vivo imaging of whole *fed117* animals revealed a high cytosolic presence of SPE-60 in primary spermatocytes, while its abundance appeared markedly reduced in secondary spermatocytes, spermatids, and spermatozoa (Fig. 4A), suggesting identical phenotypes in both sexes. To resolve its subcellular distribution in greater detail during individual developmental stages, we isolated sperm from *fed117* males and localised SPE-60::GFP in both spermatids and in vitro-activated spermatozoa (Pronase, 200 µg/ml) (Fig. 4B-E). Remarkably, our analysis revealed two distinct, stage-specific distribution patterns.

In male primary spermatocytes (Fig. 4B), SPE-60 exhibited a uniform cytosolic distribution that was slightly polarised in budding spermatids (Fig. 4C). In contrast, in spermatids and spermatozoa isolated from males, it localised to discrete cytoplasmic compartments positioned proximal to the plasma membrane (Fig. 4D, E), a spatial organisation closely resembling that MO, specialised structures known to play a role in sperm activation and fertilisation^6^.

Collectively, these findings demonstrate that SPE-60 is present throughout all phases of sperm development, from spermatocytes to mature spermatozoa. Moreover, the data reveal that both the abundance and subcellular localisation of SPE-60 undergo dynamic changes during spermatogenesis, suggesting a dual role in sperm development (spermatogenesis) and function (spermiogenesis).

### SPE-60 is required for spermatogenesis

To investigate the role of SPE-60 in spermatogenesis, we assessed sperm production and differentiation in wild-type and *spe-60(null)* mutants.

**Fig. 5:**
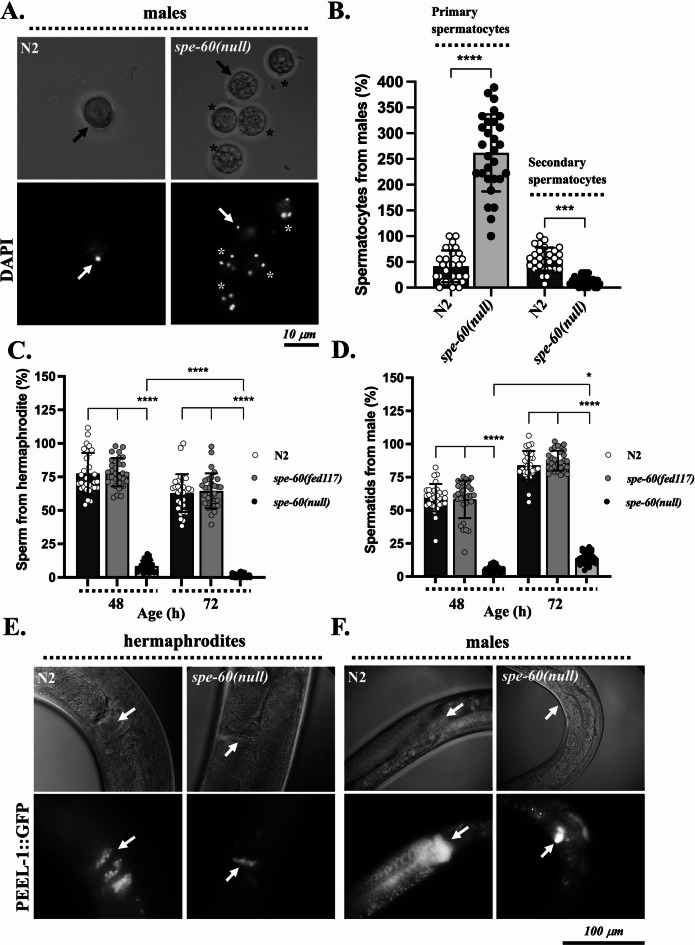
SPE-60 is required for the meiotic differentiation of primary spermatocytes. **(A)** DIC and DAPI images of primary spermatocytes isolated from *spe-60(null)* and N2 wild-type males. Black and white arrows indicate the positions of DAPI stained DNA in nuclei looking normal. Asterisks indicate terminal *spe-60(null)* spermatocytes arrested in abnormal meiotic states with multiple chromosome clusters. **(B)** The amounts of primary and secondary spermatocytes isolated from *spe-60(null)* mutant males compared to those of N2 wild-type males. **(C**,** D)** The amounts of sperm isolated from *spe-60(null)* hermaphrodites **(A)** and males **(B)** compared to N2 wild-types. **(E**,** F)** Differential interference contrast (DIC) and in vivo fluorescence microscopy images of young adult hermaphrodites **(E)** and males **(F)** expressing a GFP-tagged PEEL-1 fusion protein under control of the native *peel-1* promoter. N2 wild-types (WT) and *spe-60(null)* mutants are shown for comparison. White arrows indicate the position of GFP-labelled sperm within the animals. The values represent the means (± SD) of *N* = 3 independent experiments involving *n* ≥ 10 animals per trial. *p < 0.0332; **p < 0.0021; ***p < 0.0002; ****p < 0.0001 (ordinary one-way ANOVA).

Quantitative analysis at 48 (early adulthood) and 72 h post-hatching (adulthood) revealed a profound reduction in spermatocyte meiotic differentiation in *spe-60(null)* animals relative to age-matched wild-type controls. DAPI staining of *spe-60(null)* primary spermatocytes isolated from Males revealed abnormal chromosome distributions at 20 °C, as well as an arrest in development without undergoing meiotic divisions or cytokinesis (Fig. 5A). The failure of meiosis resulted in an accumulation of primary spermatocytes (Fig. 5B), which showed 3 to 5 distinct chromatin clusters at the terminal stage (Fig. 5A; Fig. S4 A, C). This resulted in a significant reduction in the number of secondary spermatocytes (Fig. 5B) and consequently of spermatids. Conversely, wild-type primary spermatocytes predominantly exhibited a single condensed karyosome cluster (Fig. 5A) before undergoing meiotic divisions^25,58^ (Fig. S4 B, C).

Young adult *spe-60(null)* hermaphrodites exhibited an 85% decrease in total sperm production, with a further decline during adulthood, that was drastically more pronounced than in wild-types (Fig. 5C, E). Similarly, *spe-60(null)* Males showed a 96% reduction in sperm abundance during early adulthood and 86% at the adult stage (Fig. 5D, F). However, male mutants partially retained the capacity to accumulate limited numbers of spermatids over time, indicating partial progression through spermatogenesis.

These observations were supported by in vivo DIC and fluorescence microscopy using a PEEL-1::GFP reporter (*oxSi87*) under the native *peel-1* promoter, which effectively highlighted sperm cells in both *spe-60(null)* and wild-type hermaphrodites and males (Fig. 5E, F). The data demonstrate that loss of SPE-60 impairs both spermatocyte production and their progression into spermatids. Nonetheless, a subset of *spe-60(null)* spermatocytes completed differentiation, as evidenced by the occasional presence of fertilized eggs on assay plates (Figs. 1B, 2C and 3A).

**Fig. 6:**
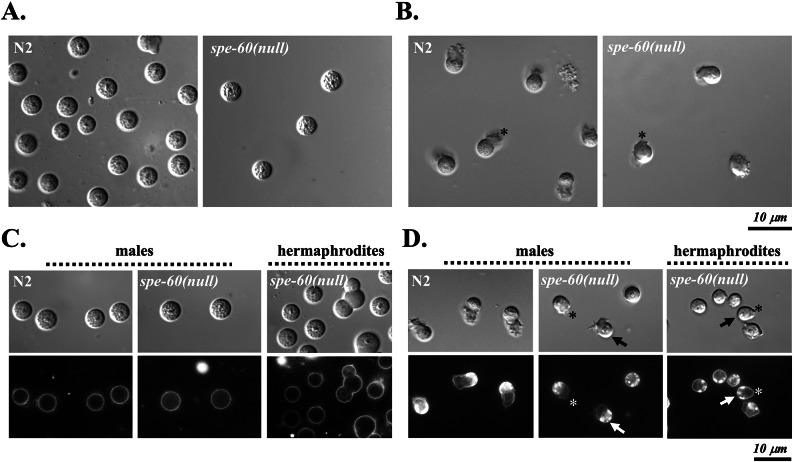
*spe-60(null)* spermatozoa display abnormal pseudopod morphogenesis but normal MO fusion. **(A**,** B)** DIC images of spermatids **(A)** and in vitro activated spermatozoa **(B)** isolated from N2 wild-types and *spe-60(null)* mutant males. Asterisks denote pseudopods, which are markedly shorter in *spe-60(null)* spermatozoa compared to the N2 wild-type. **(C**,** D)** DIC and fluorescence microscopy images of FM 1-43 labelled spermatids **(C)** and spermatozoa **(D)** from wild-type and *spe-60(null)* hermaphrodites and males. In male spermatids, activation was induced using 200 µg/ml Pronase in the presence of the lipophilic dye FM 1–43, which labels the plasma membrane (PM) and reveals MO-PM fusion. Spermatids exhibit a spherical morphology with uniform PM staining, while activated spermatozoa display specific pore structures (bright puncta, arrows) on the sperm head. The disrupted pseudopod structures exhibited by *spe-60(null)* mutants are indicated by asterisks.

To evaluate the role of SPE-60 in spermiogenesis, we analysed the morphology and motility of spermatids and in vitro-activated spermatozoa from wild-type and *spe-60(null)* males. DIC microscopy revealed that spermatid morphology was normal in *spe-60(null)* mutants (Fig. 6A). However, mutant spermatozoa failed to extend normal pseudopods and were immotile, despite exposure to various activation stimuli described in literature, including Pronase, Proteinase K, Triethanolamine, Zinc-chloride, and Monensin (Tab. S1). The pseudopods exhibited by *spe-60(null)* male spermatozoa were markedly shorter and morphologically abnormal compared to wild-type controls (Fig. 6B). Identical defects were also observed in sperm isolated from *spe-60(null)* mutant hermaphrodites.

In addition, to assess whether MO fusion with the plasma membrane, a hallmark of spermatid activation, is disrupted in *spe-60(null)* mutants, we used the lipophilic membrane dye FM1-43 to visualise fusion pore formation. As expected, no fusion pores were detected in either wild-type or *spe-60(null)* spermatids (Fig. 6C). However, activated *spe-60(null)* spermatozoa displayed fusion pores comparable to those of wild type, indicating that the fusion occurred normally (Fig. 6D).

While all tested in vitro activators successfully triggered MO fusion in both wild-type and *spe-60(null)* spermatozoa, none induced proper pseudopod extension in the mutants. Nevertheless, approximately 1 in 30 spermatozoa from mutant hermaphrodites and males exhibited wild-type-like morphology and motility, suggesting residual functional capacity.

In summary, these results reveal a dual role for SPE-60, which is required in both pre- and post-meiotic development. While it is critical for efficient spermatocyte differentiation, its loss additionally impairs sperm activation by disrupting pseudopod extension, however, without affecting MO fusion. This is characteristic for both sexes.

### SPE-60 colocalises with membranous organelles during spermiogenesis

In the light of the sharply organised subcellular localisation pattern of SPE-60 through spermatogenesis and its striking resemblance to MOs^6^ in spermatids and spermatozoa, we examined its potential association with MOs in spermatocytes, spermatids, and spermatozoa using immunostaining on *spe-60(fed117)* males that express an integrated SPE-60::GFP fusion under the native *spe-60* promoter, using specific antibodies against MOs (1CB4) and GFP (GF28R).

**Fig. 7:**
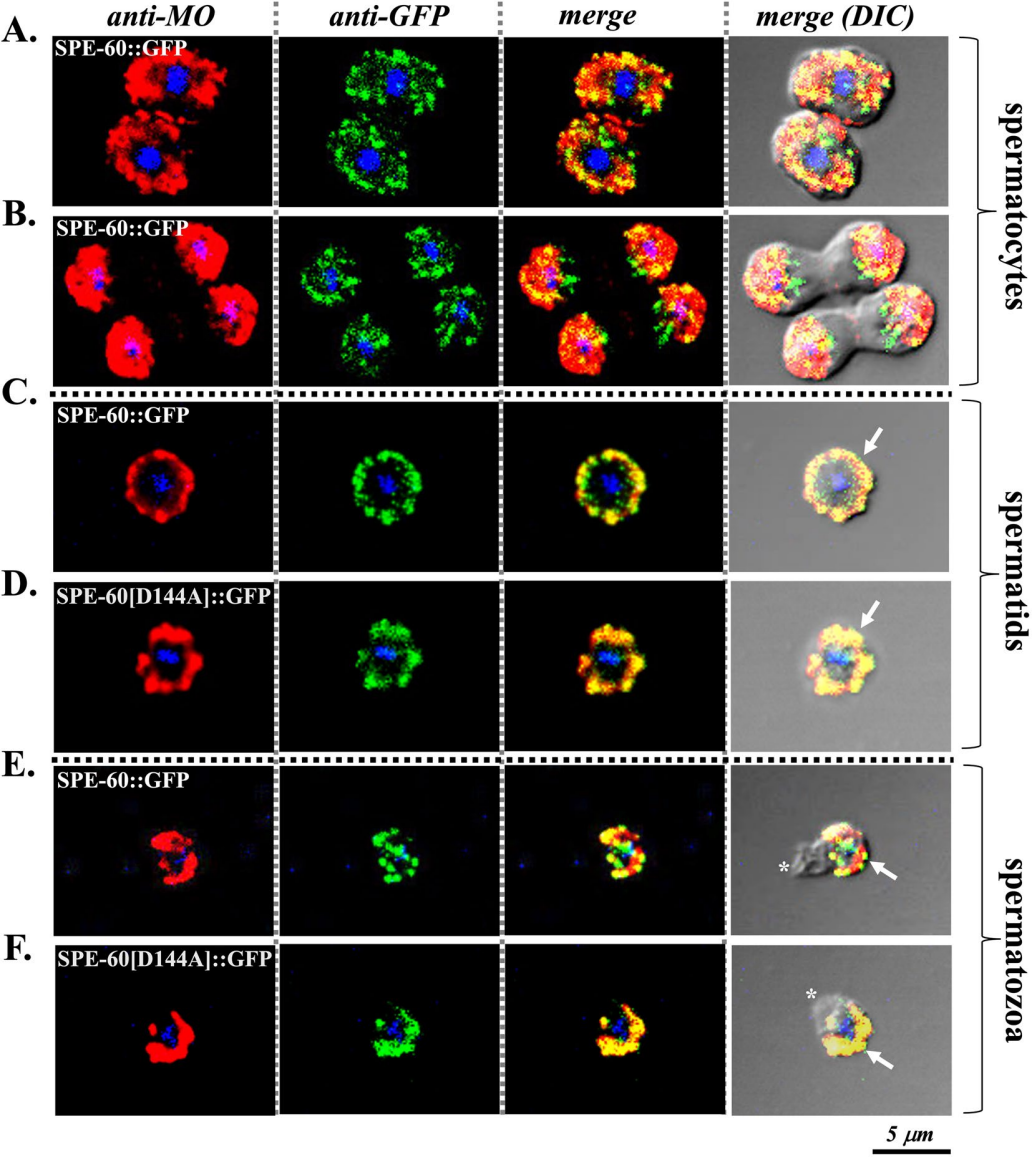
Sub cellular colocalisation of SPE-60 and MOs during sperm development. DIC and fluorescence images of co-immunostained SPE-60 and MOs illustrating the presence of SPE-60 during spermatogenesis in *spe-60(fed117)* expressing SPE-60::GFP and *spe-60*[D144A] mutants expressing SPE-60[D144A]::GFP. Antibody-mediated colocalisation (see Methods section) suggests that SPE-60 (green, anti-GFP) and MO, (red, anti-MO 1CB4) occasionally colocalise in **(A)** spermatocytes and **(B)** budding spermatids due to a rather random cytosolic distribution. In contrast, SPE-60 and MOs are relocated and closely associated (yellow) in both wild-type **(C)** and mutant spermatids **(D)** (white arrows in merged images). This association persists in spermatozoa **(E**,** F)** (white arrows). Nuclear DNA is labeled with DAPI (blue). Asterisks indicate the pseudopods of spermatozoa.

Thus, we found that immunostained SPE-60 exhibited an unclear colocalisation with fibrous body membranous organelles (FB-MOs) in spermatocytes and budding spermatids (Fig. 7A, B) and a highly precise colocalisation with MOs in both spermatids (Fig. 7C) and spermatozoa (Fig. 7E) of *fed117* males. Importantly, this spatial association was also maintained in the kinase-deficient *spe-60*[D144A] mutants harbouring the D144A substitution in the HRD motif (Fig. 7D, F). These results indicate that SPE-60 physically associates with MOs during spermiogenesis independently of its enzymatic function.

Taken together, our findings on male sperm suggest that SPE-60 is a structural component of MOs, potentially contributing to their organisation or particular function during spermiogenesis, despite not being required for the process of MO fusion.

### SPE-60 acts downstream of SPE-6 to mediate pseudopod extension during sperm activation

Sperm activation in *C. elegans* is regulated by two sex-specific signaling pathways—the hermaphrodite-specific SPE-8 and the male TRY-5 pathway, both of which converge on the casein kinase SPE-6^34,55,59^. To determine whether SPE-60 functions within or downstream of this axis, we generated *spe-6(hc163)*,*spe-60(null)* double mutants following genetic epistasis.

**Fig. 8:**
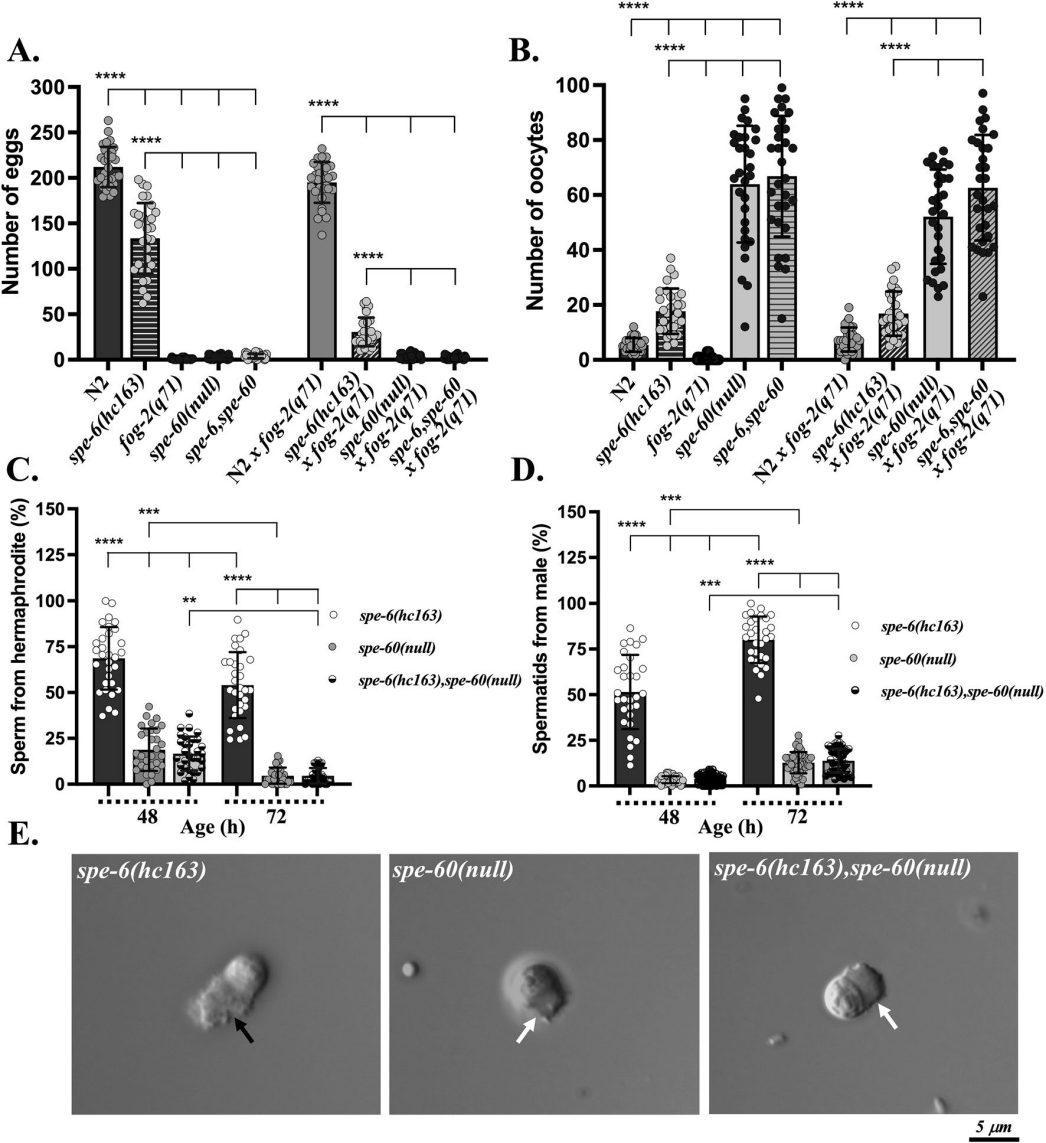
*spe-6(hc163)*,*spe-60(null)* double mutant sperm phenocopies *spe-60(null)*. The *spe-60(null)* single and *spe-6(hc163)*,*spe-60(null)* double mutants exhibit similar numbers of eggs, oocytes, and sperm. These are distinct from those observed in *spe-6(hc163)* single mutants. **(A**,** B)** The numbers of self-progeny, cross-progeny **(A)**, and oocytes **(B)** produced by single and double mutant hermaphrodites and males. **(C**,** D)** The ratios of sperm produced by *spe-60(null)* and *spe-6(hc163)*,*spe-60(null)* hermaphrodites **(C)** and males **(D)** are presented in relation to *spe-6(hc163)* animals (48 and 72 h after hatching). The data represent the means (± SD) of *N* = 3 independent experiments involving *n* ≥10 animals per trial. *p < 0.0332; **p < 0.0021; ***p < 0.0002; ****p < 0.0001 (ordinary one-way ANOVA). **(E)** DIC images of prematurely activated spermatozoa isolated from *spe-6(hc163)* and *spe-6(hc163)*,*spe-60(null)* males, compared with in vitro activated spermatozoa from *spe-60(null)* are depicted. Pseudopods of *spe-6(hc163)* spermatozoa (black arrow) exhibit normal morphology, compared to of *spe-60(null)* and *spe-6(hc163)*,*spe-60(null)* (white arrows).

Noteworthy, we found that the double mutants phenocopied the *spe-60(null)* single mutants, showing nearly complete sterility. Egg and oocyte counts on assay plates revealed that *spe-6*,*spe-60* double mutant hermaphrodites laid only 7 ± 5 eggs and 67 ± 23 oocytes, closely resembling the sterility phenotype of *spe-60(null)* and markedly differing from *spe-6(hc163)* hermaphrodites (107 ± 29 eggs, 18 ± 8 oocytes) (Fig. 8A, B). Furthermore, similar to *spe-60(null)* males, *spe-6(hc163)*,*spe-60(null)* double mutant males exhibited impaired cross-fertility when mated to *fog-2(q71)* spermless hermaphrodites, generating significantly fewer eggs and higher numbers of unfertilised oocytes than *spe-6(hc163)* males.

Additionally, sperm quantification in age matched young adult and adult worms (48- and 72-hours post-hatching, respectively) revealed that double mutants generated sperm numbers similar to *spe-60(null)* animals, showing reductions over time, whereas *spe-6(hc163)* mutants were able to maintain higher sperm levels (Fig. 8C, D). Sperm storage declined substantially in both *spe-60(null)* and *spe-6*,*spe-60* hermaphrodites over time, with a 72% reduction between early and late adulthood, while this effect was less pronounced in *spe-6(hc163)* and wild-type animals. Importantly, spermatids from *spe-6*,*spe-60* males showed premature activation - a known phenotype of *spe-6(hc163)* mutants^25^ - but failed to form normal pseudopods (Fig. 8E).

Collectively, these data position SPE-60 in both sexes functionally downstream of SPE-6, where it is specifically required for pseudopod extension without affecting initial activation signalling events.

## Discussion

The nematode *Caenorhabditis elegans*, though a relatively simple model organism, harbors a complex regulatory network governing sperm development and function.

In this study, we identify *B0207.7*, a previously uncharacterised sperm gene, also expressed in neurons^60^ required for both sperm development and motility. Based on its expression pattern in sperm and phenotypic impact, and in line with established nomenclature^61^ we have designated this gene as *spe-60*. Spatial transcriptomic analyses, including high-resolution RNA tomography^62^ revealed that *spe-60* expression mirrors that of other *spe* genes, supporting its inclusion in this class. Functional characterisation of *spe-60* null alleles revealed a severe reduction in spermatid production, along with a specific defect in sperm activation: although membranous organelle (MOs) fusion occurred normally, pseudopod extension was disrupted. Consequently, *spe-60(null)* mutants are subfertile, producing drastically reduced numbers of progeny, with the vast majority of oocytes remaining unfertilized. These phenotypes are characteristic of both hermaphrodites and males, suggesting that the fertility defect is caused by sperm rather than oocytes^59,61^.

Interestingly, the dual role of *spe-60* in spermatogenesis is reminiscent of two other *spe* mutants carrying loss-of-function alleles: *spe-6*^58,63^ and *spe-4*^31,32^. The casein kinase SPE-6 is essential at multiple stages of sperm development^25^ and its *null* alleles impair the FB-MO assembly during spermatogenesis, leading to developmental arrest of primary spermatocytes^58^. Notably, arrested primary spermatocytes have also been observed in *spe-60(null)* mutants. Nevertheless, in contrast to the complete arrest observed in sterile *spe-6* mutants carrying the *null* allele *hc49*^58^, *spe-60(null)* animals maintained the capacity to produce a reduced number of functional spermatids. Moreover, both proteins, wild-type SPE-6 and SPE-60 show a randomised and diffuse cytoplasmic pattern in spermatocytes, with no tightly organised subcellular localisation (Figs. 4B and 7A and B). However, whereas wild-type SPE-6 shifts to a perinuclear pattern around the chromatin in spermatids^63^ and is translocated to the pseudopod during activation, both wild-type and mutant SPE-60 persistently colocalise strongly with MOs during spermiogenesis (Fig. 7C-F, Fig. S7; Tab. S3).

Furthermore, unlike *spe-6* loss-of-function primary spermatocytes, which display a single condensed nucleus at the terminal stage of arrest, *spe-60(null)* primary spermatocytes arrest with multiple chromatin clusters (Fig. 5A, B; Fig. S4). This is very similar to the state of *spe-18* deficient spermatocytes, which arrest undivided showing defects in meiotic chromosome segregation and cytokinesis with abnormal chromosome distributions. SPE-18 has been found to localise to pre-FB complexes, where it functions beside the kinase SPE-6 to localise MSP assembly^64,65^. However, as outlined in the existing literature, the function of SPE-18 appears to be confined to meiosis. By contrast, SPE-60 fulfils an additional function in spermatogenesis as it is involved in pseudopod morphogenesis, which is essential for sperm motility.

A dual function profile comparable to that of *spe-6* is also observed in *spe-4* loss-of-function mutants. SPE-4 encodes a presenilin-like membrane protein that is essential for the organisation of the FB-MO and for the asymmetric partitioning of macromolecules during spermatogenesis^66^. Similar to *spe-6-* and *spe-60*-deficient hermaphrodites, *spe-4*-deficient animals accumulate arrested spermatocytes in the seminal vesicle. SPE-4 localises within the Golgi/ER-derived FB-MOs and segregates with spermatids as they leave the residual body during spermatogenesis, and loss-of-function mutations in *spe-4* have been found to impair protein transfer during budding^32^. In contrast, SPE-60 does not specifically localise to FB-MOs in spermatocytes and loss of SPE-60 function does not alter cytosolic protein transfer during budding, suggesting distinct functions. However, both proteins SPE-4 and SPE-60 have been found to colocalise with MOs during spermiogenesis^31^ (Fig. 7; Fig. S7).

The phenotypes of *spe-60-*deficient sperm also differ morphologically from those of *spe-4* and *spe-6* during spermiogenesis. While *spe-6* and *spe-4* mutant spermatids initiate premature activation in the absence of activation triggers, *spe-60(null)* spermatids are constitutively inactive unless activated by physiological cues. However, in contrast to *spe-60(null)* spermatids, the premature activation phenotype persists in *spe-60(null); spe-6(hc163)* double mutants, supporting a model in which SPE-60 acts downstream of SPE-6 to promote pseudopod extension, a prerequisite for sperm motility (Fig. S5). Furthermore, the distribution patterns of SPE-6, SPE-4 and SPE-60 in spermatozoa are different. While SPE-60 and SPE-4 colocalise with the MOs (Fig. 7; Fig. S7), SPE-6 is packed into the pseudopod where it colocalizes with the MSP^63^.

In *C. elegans*, the transfer of male-derived sperm to the spermatheca typically occurs, displacing the sperm of the hermaphrodite and thereby ensuring a fertilisation advantage^40^. While *spe-60(null)* male sperm are successfully transferred to *fog-2(q71)* hermaphrodites, they fail to persist beyond 24 h, unable to outcompete hermaphrodite sperm (Figs. 3A and B and 8A and B; Fig. S2). GFP-tagged kinase-deficient *spe-60*[D144A] sperm revealed a complete lack of long-term residency and motility, likely due to the stubby, immotile pseudopods observed in both sexes. All characteristics of *spe-60(null)* spermatozoa observed in vivo were confirmed in vitro using multiple activators (Tab. S1), emphasising the consistency of the observed defects across experimental conditions. Nonetheless, the potential exists for the effect of *spe-60(null)* mutations on spermiogenesis to be merely an indirect consequence of its earlier influence on spermatocyte differentiation. Defective spermatocytes may simply produce spermatids that are unable to form normal pseudopods during spermiogenesis. This has the potential to alter the process of fertilisation because the pseudopod, which carries MO-derived proteins that are essential for fertilisation (e.g. SPE-9, SPE-38) and are translocated to its surface after MO fusion, is the site where the interaction between the spermatozoon and the oocyte takes place^36,39^. It is notably that *spe-60(null)* spermatozoa are capable of fertilisation despite having abnormally short immotile pseudopods, however, the number of resulting progenies is drastically reduced. This suggests a defect in the ability to reach and contact the oocyte rather than a defect in the process of fertilisation itself (Figs. 1B and C, 2C and D and 3A and B).

The activation defects described partially resemble those in *fer-1(hc1ts)* mutants, which exhibit both disrupted MO fusion and deficient pseudopod extension^33^. The FER-1 protein, a member of the Ferlin family, has been shown to be associated with MOs during spermiogenesis and is considered to be essential for sperm activation. However, in contrast to *fer-1(hc1ts)*, the fusion of MOs with the plasma membrane remains intact in *spe-60(null)* spermatozoa, implicating SPE-60 in pseudopod morphogenesis acting downstream of FER-1, specifically facilitating cytoskeletal remodelling that requires MO fusion (Fig. S5). Thus, we propose a model in which SPE-60 is constitutively inactive prior to sperm activation, with MOs fusion being required for normal pseudopod morphogenesis (Fig. S7C, D). The ability of some *spe-60(null)* mutant spermatozoa to extend normal pseudopods and fertilise despite the *null* mutation (Figs. 1B, 2C and 3A) may indicate the presence of at least one factor that is constitutively activated downstream of SPE-60, or the presence of additional factors that act independently of SPE-60.

Functional dissection of SPE-60 revealed a dependence on the conserved kinase HRD motif. In particular, substitution of the *Asp* (D144) residue resulted in severe fertility defects, highlighting the essential role of this motif in kinase function. Interestingly, comparative proteomics analyses between the sperm kinases SPE-8, SPE-6, and SPE-60 show that SPE-8 and SPE-60 possess the full HRD motif, while SPE-6 lacks the catalytic *His* (H142), which may explain differences in regulation and activity (Fig. S5). Moreover, the HRD-*Arg* residue is often associated with allosteric regulation, suggesting that the SPE-60 kinase—like human ABL, JAK or drosophila SRC kinases—may undergo allosteric coupling between regulatory and active sites during sperm development^67,68^.

In conclusion, this study identifies SPE-60 as a dual-function HRD-motif kinase essential for both sperm development and function in *C. elegans*. Its dynamic presence during spermatogenesis and function in mediating pseudopod morphogenesis downstream of SPE-6, SPE-4, and FER-1 place it at a pivotal juncture within the canonical sperm activation pathway, supporting a theory in which pseudopod morphogenesis is mediated by MOs. The evolutionary conservation of its functional motifs underscores its potential relevance in higher organisms, supported by parallels to kinases implicated in cytoskeletal dynamics and human diseases such as asthenozoospermia, Alzheimer’s disease, and autoimmune disorders^14,47,48,69–71^.

Moreover, it is worth noting that two independent suppressor screens were additionally performed in the present study to confirm STRING interactions or detect unambiguous interactions with other genes. However, the results were ultimately inconclusive (data not included). Nevertheless, future studies should aim to analyse the regulatory sites of SPE-60 in greater detail, as well as elucidating putative targets in sperm and neurons. This could potentially unveil novel regulatory mechanisms linking fertility and neural health.

## Methods

### *C. elegans* strains and culture conditions

All *Caenorhabditis elegans* strains were maintained under standard laboratory conditions on nematode growth medium (NGM) agar plates seeded with *Escherichia coli* OP50 at 20 °C, following established protocols^26^. The canonical wild-type strain N2 (Bristol) served as the reference background for all comparative analyses.

Mutant and transgenic strains used in this study were obtained from the *Caenorhabditis* Genetics Center (CGC) and the National Bioresource Project (NBRP), unless otherwise specified. The strains included: VC3266 *B0207.7(gk3194)I*, BA984 *spe-6(hc163)*,*dpy-18(e364)III*, CB4108 *fog-2(q71)V*, and EG5801 *oxSi87II.* The *oxSi87* contains [*peel-1p*::N-terminal 12 amino acids of PEEL-1::GFP::*peel-1* 3’UTR + Cbr-*unc-119*(+)] II to drive GFP expression in the spermatogenic germline^72^. The *tm5353* deletion allele was acquired from the National Bioresource Program (NBRP).

The CRISPR/Cas9-engineered wild-type strains/alleles of *spe-60* were manufactured by SUNY BIOTECH Co., Ltd. as PHX4036: B0207.7(syb4036)/+ (= *spe-60(fed118))*, PHX3837: B0207.7(syb3837) (= *spe-60(fed117))*, and PHX8335: B0207.7(syb7743) I/hT2 [bli-4(e937) let-?(q782) qIs48] (I; III) (= *spe-60(fed142))*. The functional marker alleles were integrated into the *spe-60* endogenous locus. These include:

- C-terminal GFP-tagged translational fusion with *spe-60* (allele *fed117*).
- GFP-tagged functional mutations affecting the entire genomic locus (deletion allele *fed118*).
- C-terminal GFP-tagged *spe-60* variant carrying a point mutation (D144A) affecting the catalytic loop HRD motif of the kinase domain (allele *fed142*).

In *fed118*, the *spe-60* genomic sequence was replaced by GFP under the pharynx-specific *myo-2* promoter. All mutant strains were backcrossed at least four times to the N2 wild-type background to eliminate interfering background mutations and ensure phenotypic consistency. Sterile *spe-60* mutant lines were maintained using the reciprocal translocation *hT2[bli-4(e937)let-?(q782)qIs48] (I; III)*, which carries a *myo-2p*::GFP marker enabling the selection of heterozygotes by pharyngeal fluorescence^73^.

Double mutant strains were generated by conventional genetic crosses and confirmed by PCR genotyping followed by sequencing of the relevant genomic regions. All experiments were conducted on synchronised *C. elegans* populations grown under *ad libitum* feeding at 20 °C to ensure developmental consistency across genotypes.

### Fertility assays

To evaluate male fertility, synchronised L4-stage Males were cultured for 48 h 35 mm on NGM agar plates under *ad libitum* feeding at 20 °C. Mating assays were performed using six adult males and two virgin *fog-2(q71)* hermaphrodites per plate. After overnight mating, inseminated hermaphrodites were isolated, transferred daily to fresh plates until egg-laying ceased, and their progeny were counted at the L4 or young adult stage (48 h post-hatching). Control experiments were conducted using wild-type N2 Males. Control Males were generated using heat shock on 5 L4 hermaphrodite larvae, which were incubated for 5 h at 30 °C under *ad libitum* feeding on 35 mm plates seeded with *E. coli* OP50. This was followed by a recovery period at 20 °C, after which Male progeny was selected and Maintained by crossing with hermaphrodites at 20 °C. Mutant males were generated by applying standard heat shock treatment (5 h at 30 °C) to hT2-balanced L4 mutant hermaphrodites (expressing an hT2-integrated pharynx-GFP). These males were then maintained by crossing them with hermaphrodites of the same genotype. Unbalanced mutant males (with no pharynx-GFP) were selected and used for the experiments.

For hermaphrodite self-fertility assays, *spe-60(null)* L4 larvae were individually transferred to 35 mm NGM agar plates and allowed to lay eggs at 20 °C under *ad libitum* feeding. Adults were transferred daily to new plates for brood size quantification.

Sperm transfer was examined by mating *spe-60(null)* or transgenic wild-type *spe-60(fed117)* males expressing *PEEL-1::GFP (oxSi87 II)* with *fog-2(q71)* hermaphrodites. Post-mating transferred sperm were visualised using differential interference contrast (DIC) and fluorescence microscopy using a AxioImager M2 microscope (Zeiss).

### Sperm isolation and in vitro activation

Sperm activation assays were performed using celibate males and hermaphrodites with wild-type or mutant genetic backgrounds, cultured under *ad libitum* feeding conditions. L4-stage worms were transferred to 35 mm NGM agar plates and incubated at 20 °C for 48 h. For sperm isolation, adult worms were transferred to a 4.5 µL droplet of sperm medium (SM buffer; 50 mM HEPES, 45 mM NaCl, 1 mM MgSO₄, 25 mM KCl, 5 mM CaCl₂, 10 mM dextrose; pH 7.8)^74^ placed on a glass microscope slide (Thermo Scientific) pre-marked with a hydrophobic PAP pen (DAKO). Coverslips (20 × 20 mm; Carl Roth) were than applied and sealed with Vaseline.

Spermatozoa were released by mechanically dissecting the gonads using two 25-gauge needles under a stereomicroscope. For in vitro activation of Male-derived sperm, 0.5 µL of SM buffer containing either an ectopic in vitro activation trigger (e.g., Pronase) or buffer alone (control) was added to the preparation. Samples were incubated for 30 min at 20 °C prior to imaging. Pronase (2 mg/mL in SM buffer) served as the standard activator unless otherwise indicated (see Table S2). Following activation, a 20 × 20 mm glass coverslip (Carl Roth GmbH) was gently placed over the sample and sealed with Vaseline to prevent evaporation during microscopy.

Sperm activation was evaluated by differential interference contrast (DIC) and fluorescence microscopy. Individual sperm were classified based on cell morphology and the presence of MO fusion pores. Cells with spherical morphology, lacking pseudopod extension and MO fusion pores, were categorised as non-activated. Sperm with clearly visible fusion pores and motile, fully extended pseudopods—defined as projections at least equal in length to the cell body—were scored as activated^75^. Sperm showing MO fusion and pseudopods shorter than the cell body (“stubby” phenotype) were also classified as activated.

### MO fusion assay

MO fusion was assessed using a lipophilic membrane dye-based assay as previously described^33^with minor modifications. Briefly, isolated spermatids or in vitro-activated spermatozoa were incubated in sperm medium (SM buffer) containing 5 µg/mL FM 1–43 dye (Thermo Scientific) for 3 min at room temperature.

Following incubation, samples were mounted under a 20 × 20 mm glass coverslip and imaged immediately using a Zeiss laser scanning confocal microscope (LSM700) equipped with a 100-fold oil immersion objective and an AxioCam MRm camera (Zeiss). Successful MO–plasma membrane fusion was indicated by the presence of punctate FM 1–43 fluorescence localised along the plasma membrane. Cells lacking membrane-associated FM 1–43 signal were scored as nonactivated.

### Quantification of sperm activation

Sperm activation was quantified in synchronised worm populations grown at 20 °C under *ad libitum* feeding. To ensure male celibacy, L4-stage Males were Manually separated from hermaphrodites and cultured individually for 48 h. Adult males were than subsequently dissected as described above to isolate spermatozoa.

To assess activation status, isolated sperm were incubated in sperm medium (SM buffer) Supplemented with 5 µg/mL FM 1–43 (Thermo Scientific) for 3 min. The presence of pseudopod extension and membrane-localised fusion pores was used as a combined indicator of successful activation. Cells exhibiting both morphological features were scored as activated, whereas spherical cells lacking pseudopods and fusion-associated fluorescence were scored as non-activated. The extent of spontaneous (premature) activation of male sperm was categorised using established benchmarks^63^.

### Microscopy

High-resolution imaging of spermatozoa, both in vivo and in vitro, was performed using a Zeiss AxioImager M1 differential interference contrast (DIC) and fluorescence microscope equipped with a 100-fold oil immersion objective and an AxioCam MRm camera (Zeiss). For live imaging of sperm within whole-worms, 10 Males or hermaphrodites were anesthetised in 5 µL of 5 mM Levamisole and mounted on 2% agarose pads prepared on standard glass microscope slides (Thermo Scientific). Immobilised worms were immediately imaged at 20 °C.

For imaging of isolated sperm, cells were dissected from adult males or hermaphrodites and Suspended in 4.5 µL SM buffer, with or without prior in vitro activation. Samples were sealed under a 20 × 20 mm glass coverslip using Vaseline to prevent evaporation during imaging.

GFP-tagged fusion proteins were visualised *via* excitation at 488 nm using the corresponding Zeiss filter sets. Image acquisition and basic post-processing were performed using *Time-To-Live* software (Schnabel Lab, TU-Braunschweig, Germany). Images were captured as *.lurawave* files and then cropped, saved, and converted in a format suitable for publishing, such as.*jpg*,.*bmp* or.*png*.

Imaging of fixed, immunostained sperm was conducted using a Zeiss AxioImager Z2 upright microscope coupled with an LSM 700 laser scanning confocal module. Fluorescent signals were detected using sequential acquisition channels: 488 nm for green (Alexa 488, Thermo Scientific), 555 nm for red (Alexa555, Thermo Scientific), and 405 nm for DAPI-stained nuclei. Confocal z-stacks and multichannel images were processed and rendered using the ZEN imaging software (Zeiss). Images were captured as.*czi* files and then cropped, saved, and converted in a format suitable for publishing, such as.*jpg*,.*bmp* or.*png*.

### Immunostaining

Immunofluorescence staining of isolated sperm was conducted on poly-L-lysine-coated glass slides (Thermo Scientific). A hydrophobic barrier was drawn using a PAP pen (DAKO) to restrict sample spread. Ten adult Males were dissected in 3 µL of sperm medium (SM buffer) within the marked area to release spermatocates, spermatids, and/or spermatozoa. The released cells were fixed by adding 3 µL of 4% paraformaldehyde (PFA) in SM buffer for 30 min at 20 °C.

Following fixation, samples were washed with 20 µL of PBST (PBS Supplemented with 0.01% Tween-20; Carl Roth GmbH) and permeabilised in 20 µL of PBS containing 5% Triton X-100 (Carl Roth GmbH) for 5 min. After a second PBST wash, blocking was performed in PBST containing 0.5% BSA for 30 min at room temperature.

Primary antibody incubation was carried out for 1.5 h at room temperature using rabbit anti-GFP (1:1000; GF28R, Thermo Scientific) and mouse monoclonal anti-MO 1CB4 (1:1000)^76^diluted in PBST with 0.5% BSA. Following three 10-minute washes in PBST, samples were incubated for 30 min with Alexa Fluor 488-conjugated anti-rabbit and Alexa Fluor 555-conjugated anti-mouse secondary antibodies (Thermo Scientific), each at 1:500 dilution in PBST + 0.5% BSA. 1CB4 antibodies were kindly provided by Ralf Schnabel (Schnabel Lab, Developmental Genetics, TU Braunschweig, Germany).

After secondary incubation, slides were washed three times for 10 min each in PBST and mounted using DAKO mounting medium Supplemented with 2 ng/µL DAPI (Thermo Scientific) for nuclear counterstaining. Imaging was performed using a Zeiss AxioImager Z2 microscope equipped with an LSM 700 laser scanning confocal system, and image processing was conducted using the ZEN software (Zeiss).

### Statistical analysis

All statistical analyses were performed using *GraphPad Prism* (version 10.4.2). To assess differences between groups, we exclusively employed ordinary one-way ANOVA for multiple comparisons with *p < 0.0332; **p < 0.0021; ***p < 0.0002; ****p < 0.0001. Data are presented as mean ± standard deviation (SD) unless stated otherwise. Sample sizes (n) refer to biologically independent replicates, and no data points were excluded unless stated. No corrections for multiple comparisons were applied. To ensure reproducibility, data represent the means (± SD) of *N* = 3 independent experiments involving *n* ≥ 10 animals per trial.

## Supplementary Information

Below is the link to the electronic supplementary material.

Supplementary Material 1

## Data Availability

The raw data supporting the findings of this study are available from the corresponding author at any time upon request (E-Mail: gottschling@molprev.uni-kiel.de). Processed data can be found within the manuscript or in the supplementary information files.
