# Supplementary material for: The novel HRD motif kinase SPE-60 is required for sperm development and motility in Caenorhabditis elegans
Source: Sci Rep. 2025 Sep 15;15:32539. doi: 10.1038/s41598-025-18696-2 (PMC12436626; doi:10.1038/s41598-025-18696-2)
Supplement: Supplementary file 1 — Supplementary Material 1 [file 41598_2025_18696_MOESM1_ESM.docx]

**Supplementary figures with legends**

**Fig. S1**

**
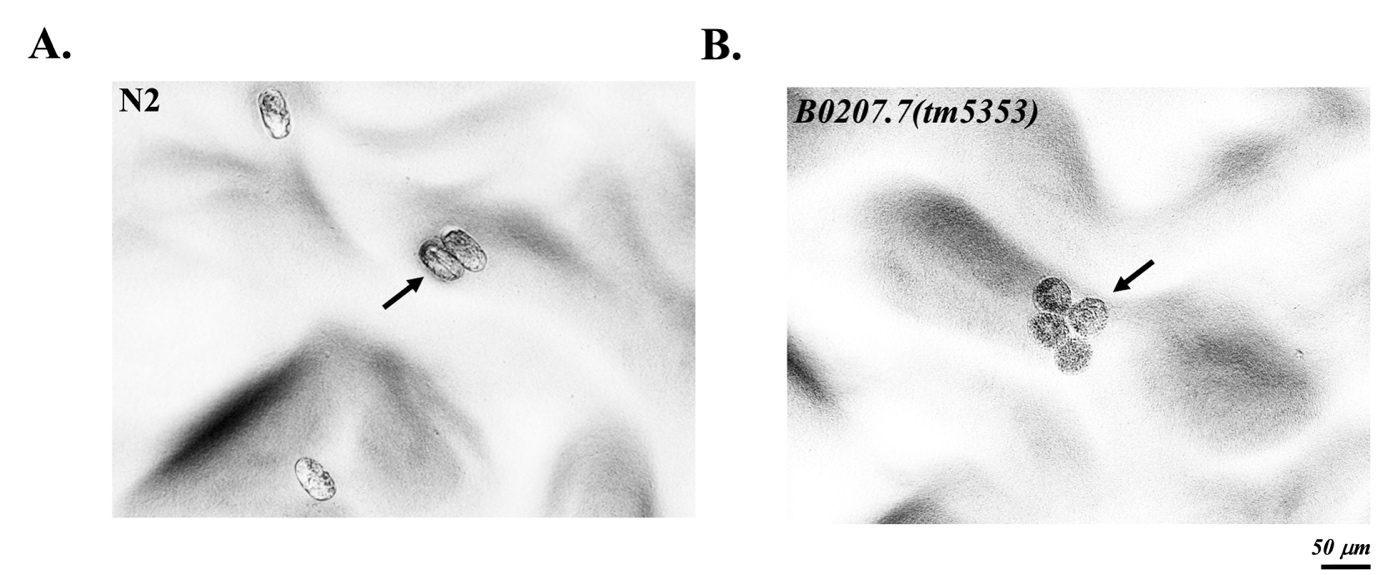
**

**Figure S1: Loss of *B0207.7* results in a sterile phenotype characterised by an excess of unfertilized oocytes.**

Bright-field microscope images of eggs and unfertilized oocytes laid by young adult hermaphrodites on agar plates seeded with *E. coli* OP50. **(A)** Wild-type (N2) hermaphrodites predominantly lay fertilized eggs. **(B)** In contrast, *B0207.7(tm5353)* loss-of-function mutant hermaphrodites lay primarily unfertilized oocytes, reflecting a sterile phenotype due to impaired self-sperm function.

**Fig. S2**

**
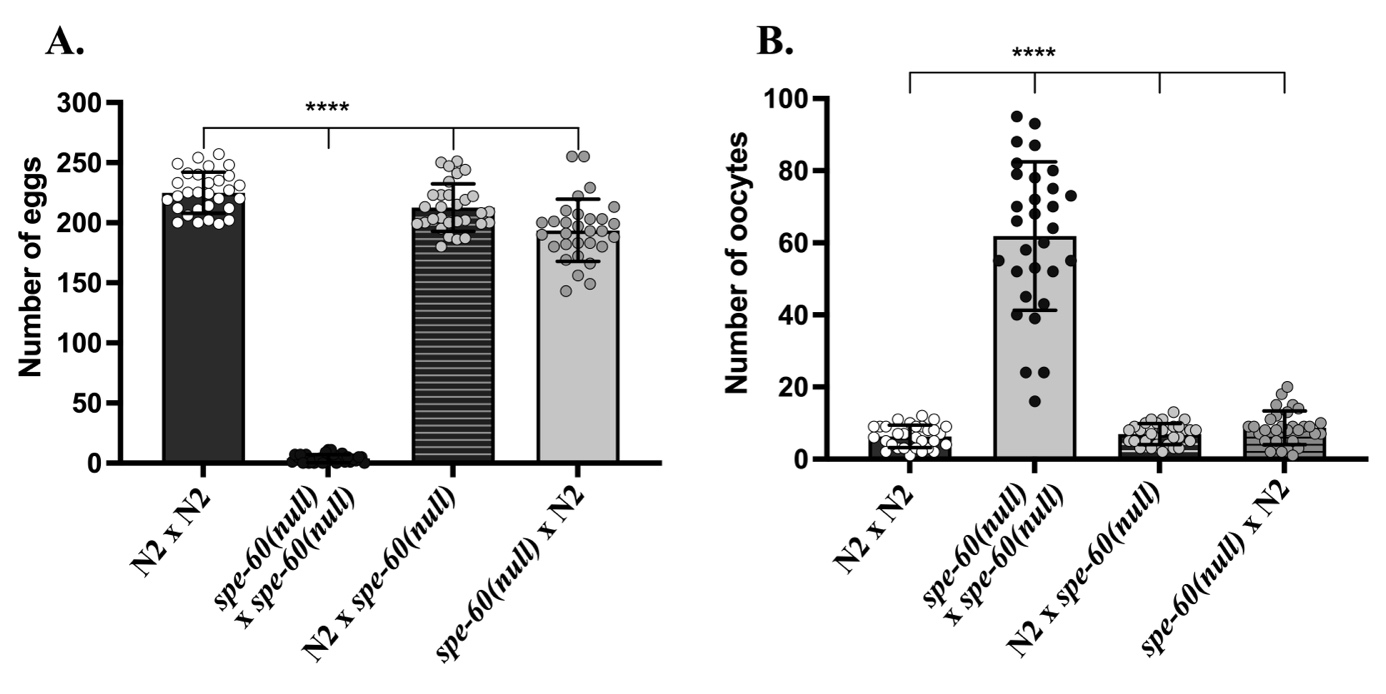
**

**Figure S2: Quantitative assessment of cross-fertility in *spe-60(null)* mutants.**

**(A, B)** Quantification of egg **(A)** and oocyte **(B)** numbers laid by *spe-60(null)* and N2 wild-type hermaphrodites following mating with either N2 wild-type or *spe-60(null)* mutant males. The values represent the means (± SD) of N = 3 independent experiments involving n ≥10 animals per trial. *p < 0.0332; **p < 0.0021; ***p < 0.0002; ****p < 0.0001 (ordinary one-way ANOVA). N2 wild-type sperm efficiently rescued the sterile phenotype of *spe-60(null)* hermaphrodites, whereas sperm from *spe-60(null)* males or hermaphrodites failed to successfully fertilize oocytes when competing with wild-type sperm. Despite this competitive disadvantage, sperm transfer from *spe-60(null)* males to both wild-type and mutant hermaphrodites was confirmed (Table S2).

**Fig. S3**

**
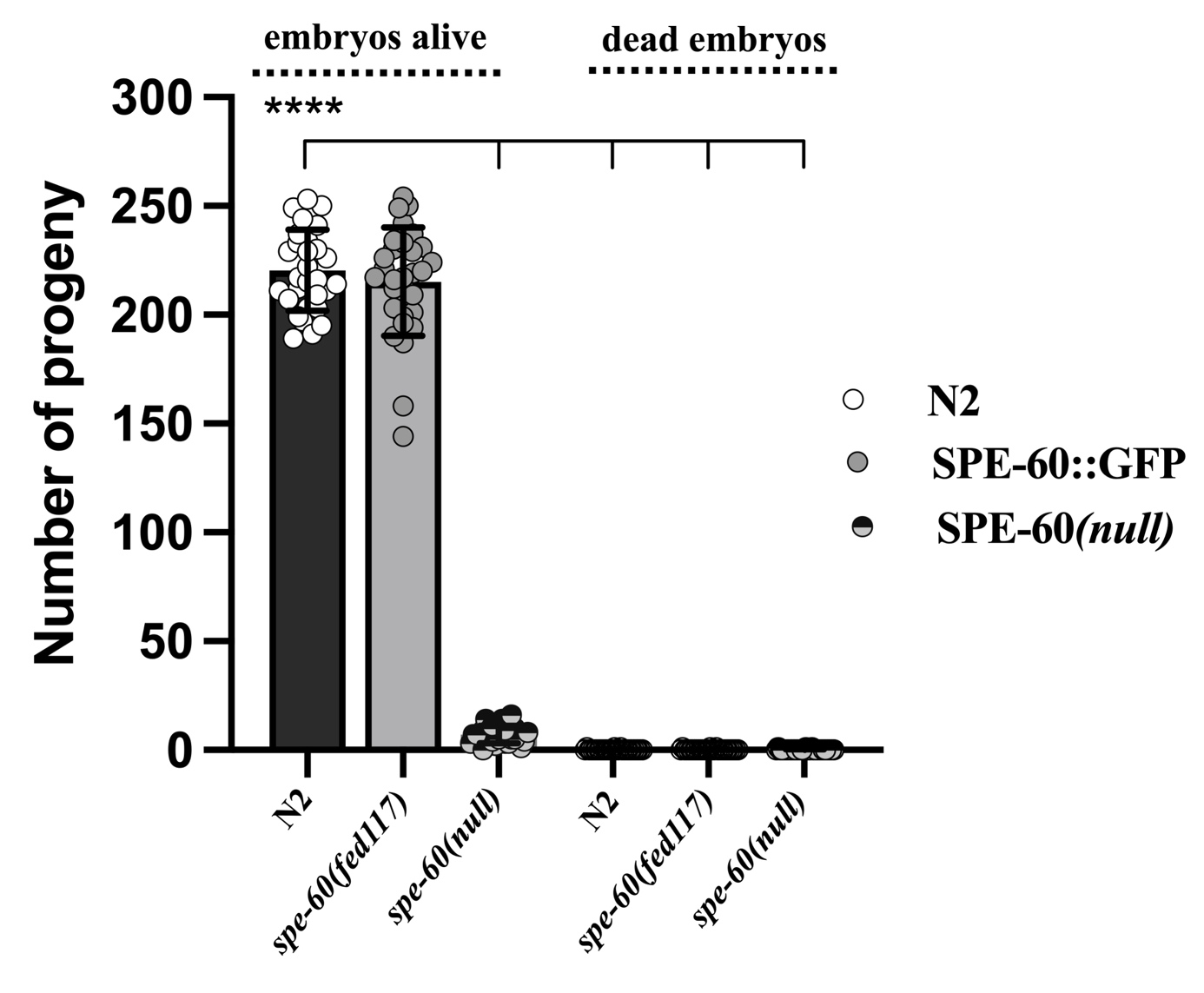
**

**Figure S3: Evaluation of embryonic lethality.**

The number of living and dead embryos produced by hermaphrodites expressing the *spe-60*::*gfp* fusion allele (*fed117)* or lacking the entire *spe-60* genomic sequence (*fed118*, *null*) was counted and compared with the number of progenies generated by the N2 wild type. No embryonic-lethal phenotypes were detected. The values represent the means (± SD) of N = 3 independent experiments involving n ≥10 animals per trial. *p < 0.0332; **p < 0.0021; ***p < 0.0002; ****p < 0.0001 (ordinary one-way ANOVA).

**Fig. S4**

**
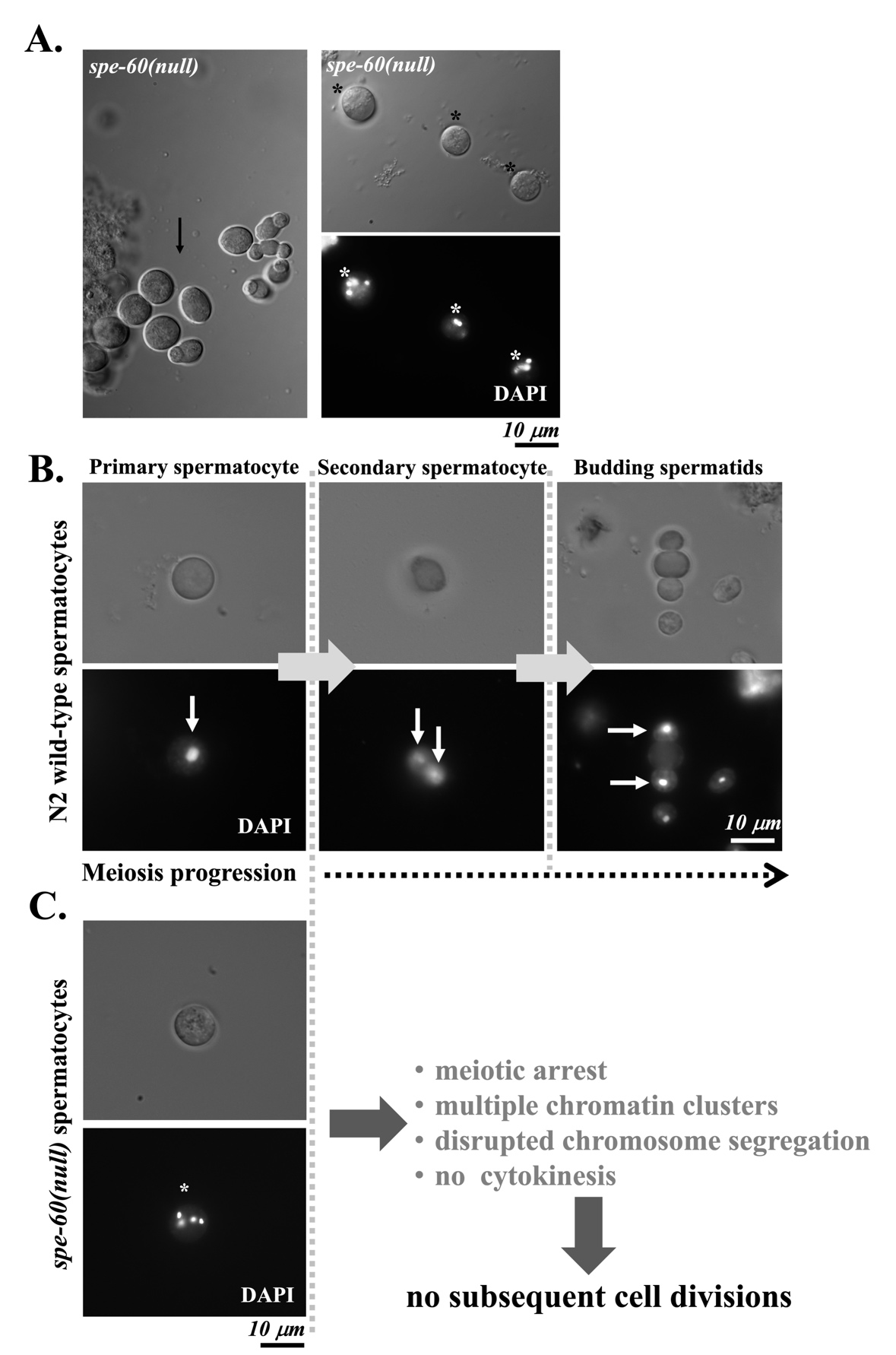
**

**Figure S4: Characteristics of meiotic differentiation in *spe-60(null)* and N2 wild-type spermatocytes.**

**A.** DIC and fluorescence images showing DAPI-stained primary spermatocytes isolated from *spe-60(null)* males. (**B, C)** Comparison of the progression of meiosis in spermatocytes isolated from wild-type N2 **(B)** and *spe-60(null)* males **(C)**.

**Fig. S5**

**
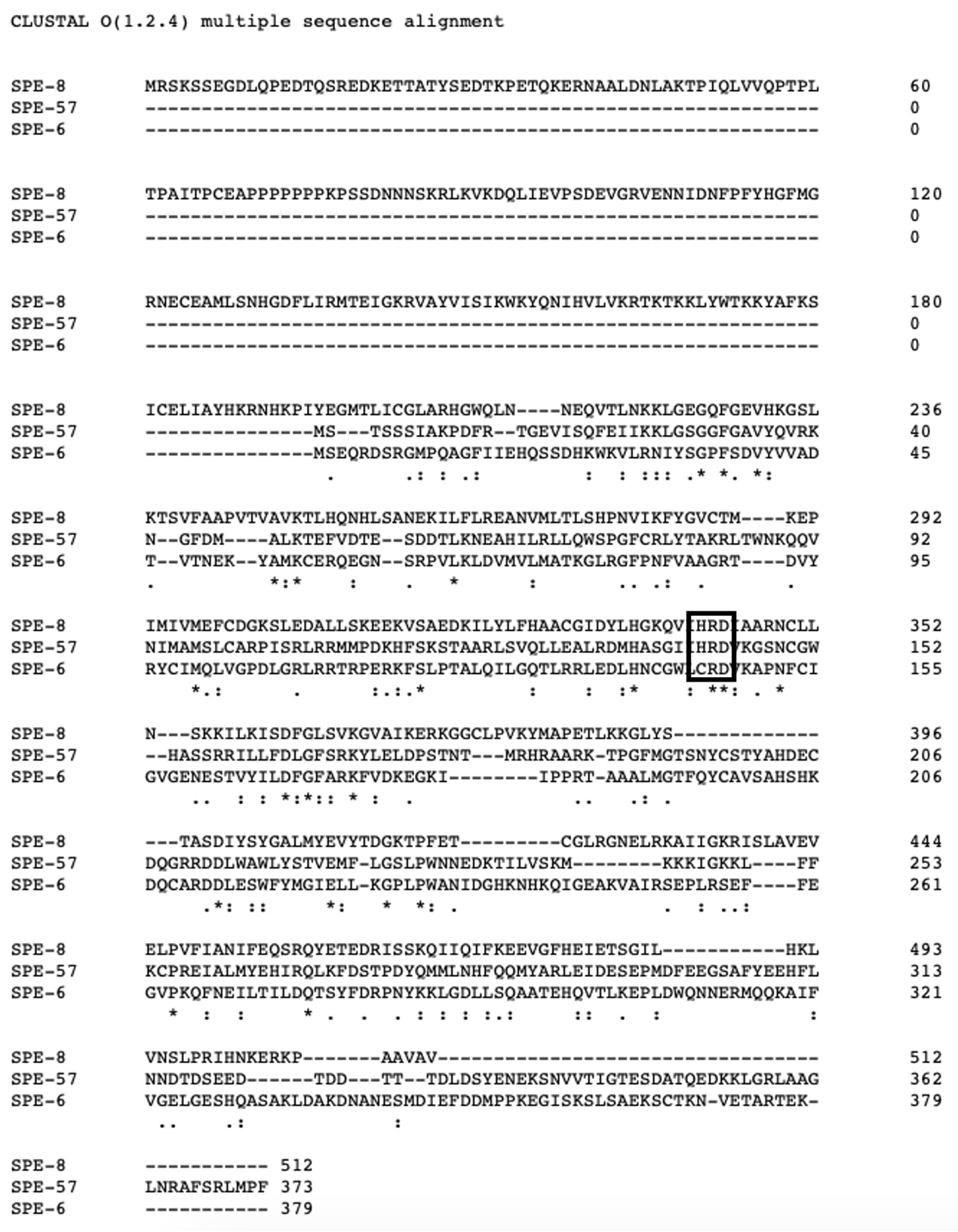
**

**Figure S5: Comparative alignment of kinase domains from SPE-8, SPE-60, and SPE-6.**

Multiple sequence alignments generated via *Clustal-Omega* illustrate conserved and divergent features within the kinase domains of the tyrosine kinase SPE-8, tau-tubulin kinase SPE-60, and casein kinase SPE-6. The conserved catalytic HRD motifs, highlighted in the black square, are present at analogous positions in SPE-8 and SPE-60 but are absent in SPE-6. Notably, the canonical histidine residue within the HRD motif is replaced by a cysteine in SPE-6, indicating divergence from the architecture of SPE-60 and SPE-6.

**Fig. S6**

**
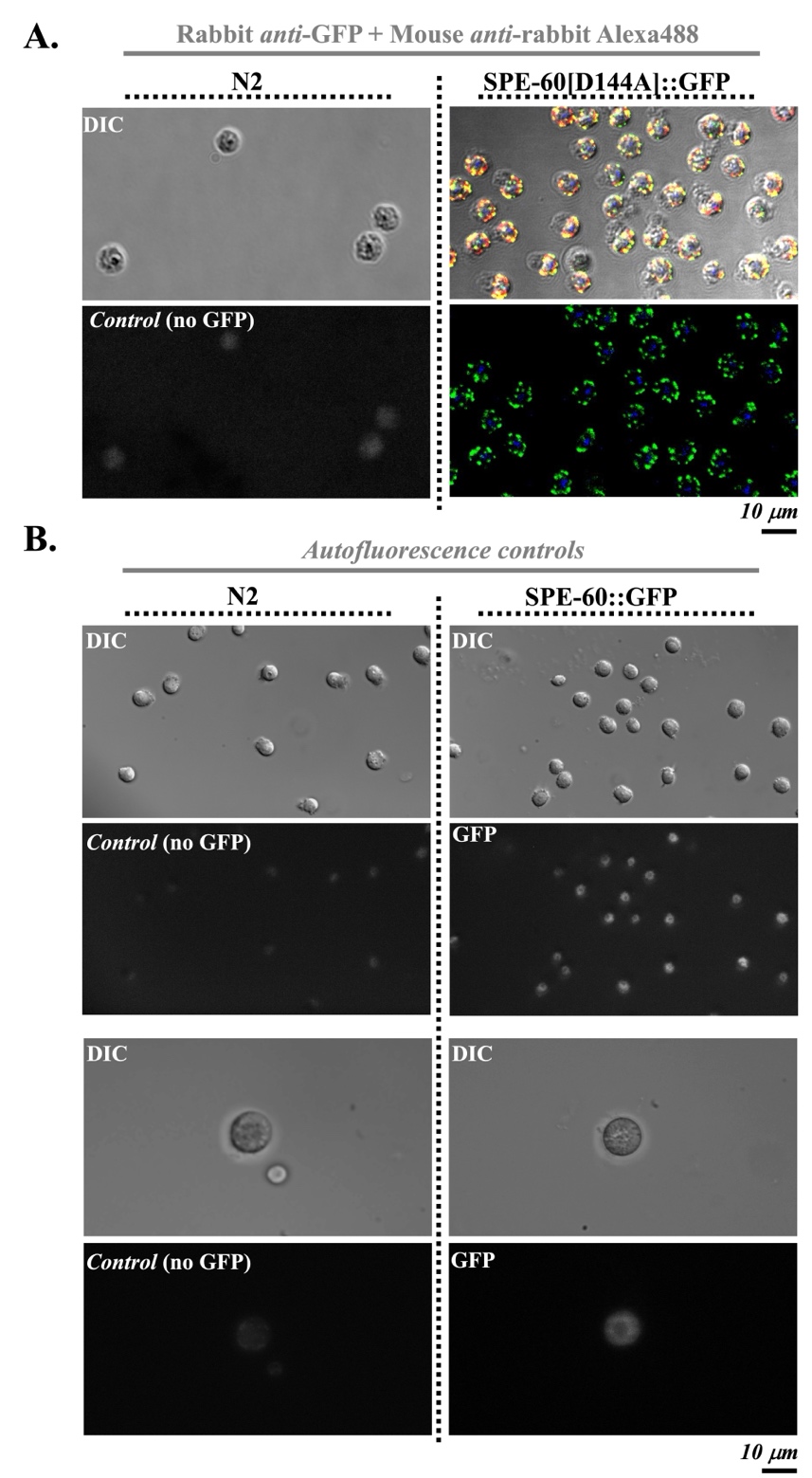
**

**Figure S6: Autofluorescence control at 488 nm (GFP channel).**

**A.** Comparison of GFP autofluorescence in sperm isolated from N2 wild-type (non-GFP control) and *spe-60* [D144A]::GFP mutant males following incubation with anti-GFP primary and Alexa Fluor 488-conjugated secondary antibodies. As indicated in the methods section, images were recorded with an LSM700 microscope (Zeiss). **B.** DIC and GFP-channel fluorescence images showing autofluorescence in spermatids and pronase-activated spermatozoa from N2 wild-type (non-GFP control) and *spe-60*[D144A]::GFP mutant males. As stated in the methods section, these images were captured using an *Axio Imager M2* microscope (Zeiss).

**Fig. S7**

**
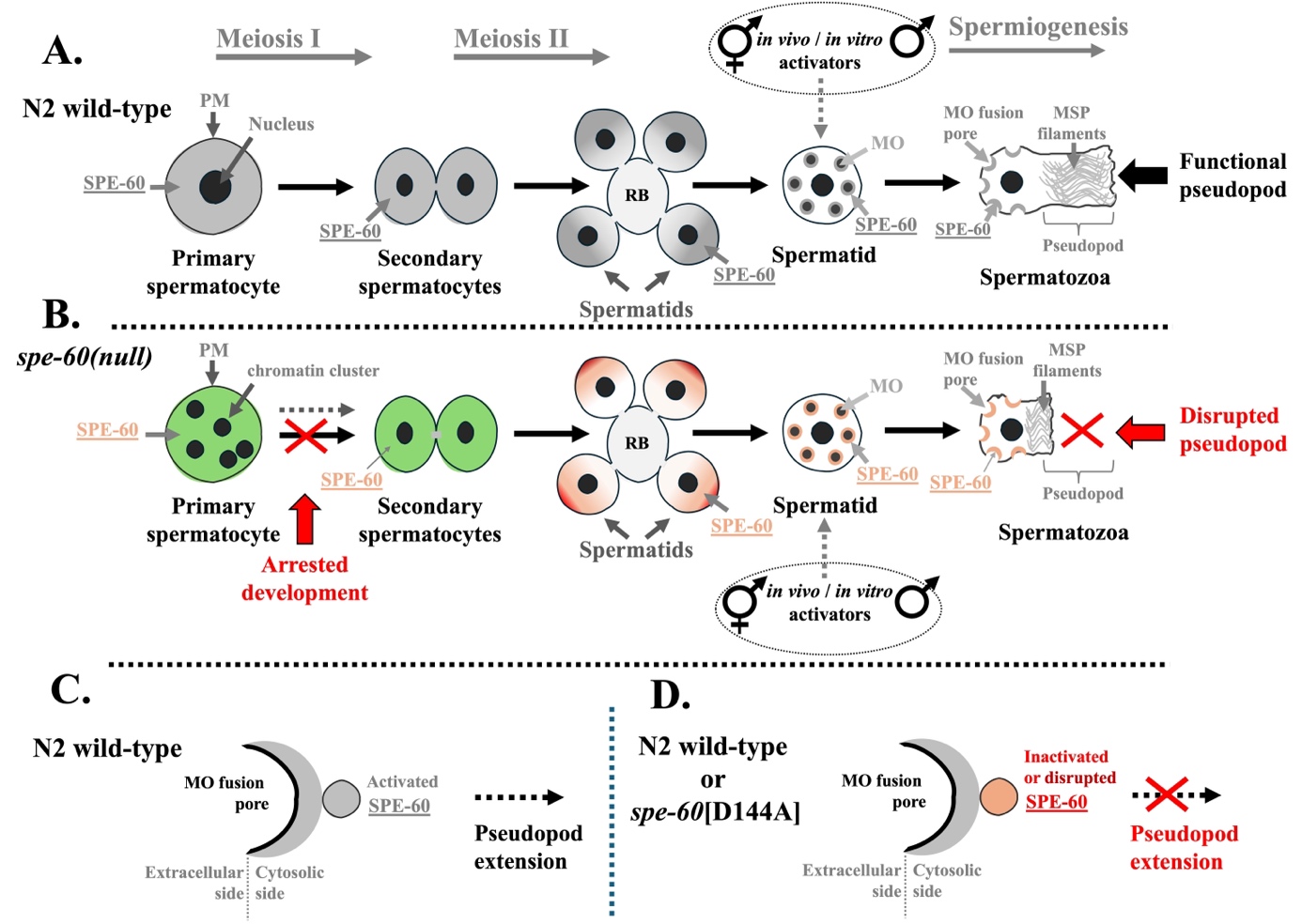
**

**Figure S7: Graphical abstract illustrating the dual function of SPE-60 in sperm development and motility.**

**(A)** Wild-type sperm from hermaphrodites and males show normal spermatocyte differentiation during spermatogenesis and normal spermatid maturation upon activation (spermiogenesis) into mature motile spermatozoa capable of fertilization. The fusion of membranous organelles (MOs) with the plasma membrane (PM) and pseudopod formation are normal. RB indicates the residual body; MSP stands for Major Sperm Protein. **(B)** In contrast, *spe-60(null)* animals are sterile. Most primary spermatocytes exhibit developmental arrest. The *spe-60(null)* primary spermatocytes that differentiate into secondary spermatocytes and then into spermatids exhibit normal development and the resulting spermatozoa exhibit normal fusion of the MOs with the PM. However, the pseudopods of these spermatozoa show disrupted morphogenesis, being stubby and immotile. Coloured shading within the cells depicts the presence and location of SPE-60 in wild-type (grey) and mutant (light red) sperm. **(C, D)** Models that show activated wild-type SPE-60 **(C)** and inactivated/disrupted wild-type or mutant SPE-60 **(D)** located at the cytosolic MO site as a member of a pathway involved in pseudopod morphogenesis.

**Supplementary tables with legends**

**Tab. S1**

**
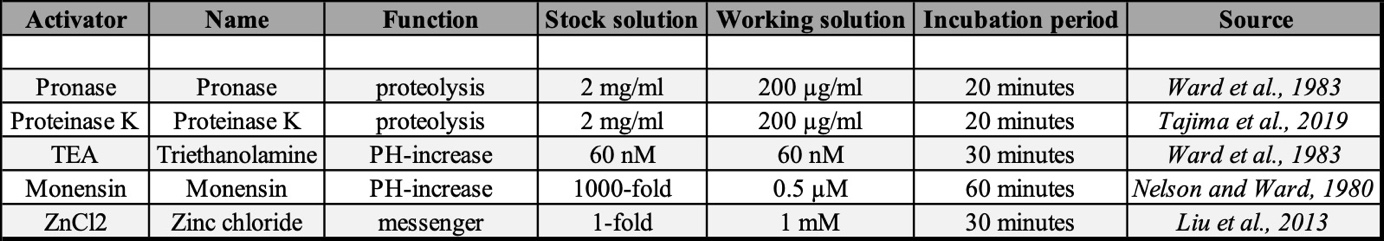
**

**Table S1: Chemical triggers used for *in vitro* spermatid activation.**

Overview of the chemical compounds applied for *in vitro* activation of *C. elegans* spermatids, including their names, molecular functions, and working concentrations or experimental conditions used in this study.

**Tab. S2**

**
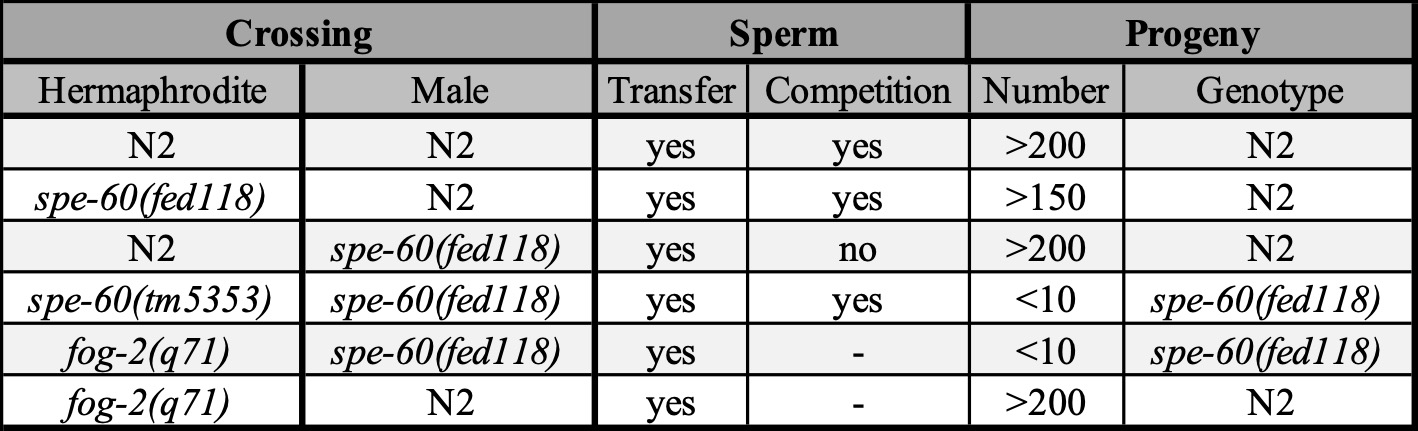
**

**Table S2: *In vivo* assessment of male fertility, sperm transfer, and competitive capacity.**

Summary of mating experiments involving wild-type and *spe-60*-deficient hermaphrodites and males conducted to evaluate the ability of males to transfer sperm and the competitiveness of their sperm in fertilization. The mating conditions and quantification criteria are detailed in the Methods section. The data represent the means ± SD from three independent experiments (N = 3), each involving ≥10 animals per condition, corresponding to the results shown in Fig. S2. For *spe-60*-deficient males, sperm transfer and successful competition were scored as “yes” if ≥ 10 F1 cross progeny exhibited pharyngeal GFP fluorescence driven by the *myo-2* promoter integrated into the *spe-60(fed118)* genetic *null* background.

**Tab. S3**

**
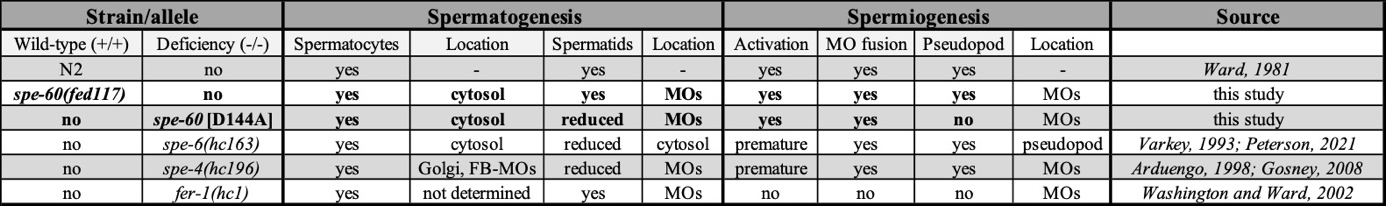
**

**Table S3: Phenotypic impact of *spe-60(null)* alleles on sperm maturation and function.**

The *spe-60*[D144A] (*fed142*) phenotypes were compared to those of the wild-type (+/+) and *spe-60(fed117)* phenotypes and to the phenotypes generated by *spe-4*, *spe-6*, and *fer-1* deficiency alleles (-/-), as discussed in the present study and in other literature sources (see the Literature section for details). This comparison focuses on spermatogenesis and spermiogenesis and includes the subcellular location, sperm activation, MO fusion, and pseudopod extension.
